# Effects of comprehensive geriatric care models on postoperative outcomes in geriatric surgical patients: a systematic review and meta-analysis

**DOI:** 10.1186/s12871-021-01337-2

**Published:** 2021-04-22

**Authors:** Aparna Saripella, Sara Wasef, Mahesh Nagappa, Sheila Riazi, Marina Englesakis, Jean Wong, Frances Chung

**Affiliations:** 1 Department of Anesthesia and Pain Medicine, Toronto Western Hospital, University Health Network, University of Toronto, MCL 2-405, 399 Bathurst St, Toronto, ON M5T2S8 Canada; 2 Department of Anesthesia and Perioperative Medicine, London Health Sciences Centre and St. Joseph Health Care, Schulich School of Medicine & Dentistry, Western University, London, ON Canada; 3 Library and Information Services, University Health Network, Toronto, ON Canada; 4 Department of Anesthesia and Pain Management, Women’s College Hospital, University of Toronto, Toronto, ON Canada

**Keywords:** Comprehensive geriatric care model, Comprehensive geriatric assessment, Surgery, Elderly, Delirium, Adverse outcomes

## Abstract

**Background:**

The elderly population is highly susceptible to develop post-operative complications after major surgeries. It is not clear whether the comprehensive geriatric care models are effective in reducing adverse events. The objective of this systematic review and meta-analysis is to determine whether the comprehensive geriatric care models improved clinical outcomes, particularly in decreasing the prevalence of delirium and length of hospital stay (LOS) in elderly surgical patients.

**Method:**

We searched Medline, PubMed, Embase, Cochrane Central Register of Controlled Trials, Cochrane Database of Systematic Reviews, Emcare Nursing, Web of Science, Scopus, CINAHL, ClinicalTrials. Gov, and ICTRP between 2009 to January 23, 2020. We included studies on geriatric care models in elderly patients (≥60 years) undergoing elective, non-cardiac high-risk surgery. The outcomes were the prevalence of delirium, LOS, rates of 30-days readmission, and 30-days mortality. We used the Cochrane Review Manager Version 5.3. to estimate the pooled Odds Ratio (OR) and Mean Difference (MD) using random effect model analysis.

**Results:**

Eleven studies were included with 2672 patients [Randomized Controlled Trials (RCTs): 4; Non-Randomized Controlled Trials (Non-RCTs): 7]. Data pooled from six studies showed that there was no significant difference in the prevalence of delirium between the intervention and control groups: 13.8% vs 15.9% (OR: 0.76; 95% CI: 0.30–1.96; *p* = 0.57). Similarly, there were no significant differences in the LOS (MD: -0.55; 95% CI: − 2.28, 1.18; *p* = 0.53), 30-day readmission (12.1% vs. 14.3%; OR: 1.09; 95% CI: 0.67–1.77; *p* = 0.73), and 30-day mortality (3.2% vs. 2.1%; OR: 1.34; 95% CI: 0.66–2.69; *p* = 0.42). The quality of evidence was very low.

**Conclusions:**

The geriatric care models involved pre-operative comprehensive geriatric assessment, and intervention tools to address cognition, frailty, and functional status. In non-cardiac high-risk surgeries, these care models did not show any significant difference in the prevalence of delirium, LOS, 30-days readmission rates, and 30-day mortality in geriatric patients. Further RCTs are warranted to evaluate these models on the postoperative outcomes.

**Trial registration:**

PROSPERO registration number - CRD42020181779.

**Supplementary Information:**

The online version contains supplementary material available at 10.1186/s12871-021-01337-2.

## Background

The current elderly population (65 years or older) is approximately 7 million in Canada, and 62 million in North America. The proportion of the elderly is increasing rapidly; 25% of the population will be 65 years or older by the year 2036 [1]. Surgery on the elderly results in greater complications, prolonged length of hospital stay (LOS), increase in emergency department visits, readmission rates, post-discharge care requirements, and health care costs [2–4]. Both delirium and frailty impede recovery post-surgery with cognitive impairment, leading to a 3-fold increase in stay in hospital and rehabilitation facilities [5–7]. Frailty with decreased physical functionality and life expectancy is associated with a 2-fold increase in postoperative complications and new physical disability [8, 9].

Comprehensive geriatric care model is a co-management program to deliver the geriatric care with personnel and expert supervision. The comprehensive geriatric care model included reduction of delirium; co-morbidity management; nutritional assessment; individualized care plan; and postoperative follow-up [10–19]. The chief constituent of the comprehensive geriatric care model is comprehensive geriatric assessment (CGA). The CGA is an established multi-domain assessment addressing patients’ physiological, social, psychological, and functional state of the elderly people [17]. Comprehensive geriatric care models consist of a multidisciplinary team comprising geriatricians, geriatric nurses, anaesthesiologists, surgeons, physiotherapists, occupational therapists, and dieticians. Enhanced recovery after surgery (ERAS) has been proven effective in decreasing LOS and morbidities of surgical patients, but they rarely have a CGA component or involvement of the geriatric team [20, 21].

Several studies had included CGA as a major component of the geriatric care model and evaluated its impact on postoperative outcomes [10, 11, 13, 15–19, 22–24]. The most common models are Proactive care of Older People undergoing Surgery (POPS) [17], Hospital Elder Life Program (HELP) pathway [14], Perioperative Optimization of Senior Health (POSH) pathway [15], person-centred care (PCC) pathway [12], Liaison Intervention in Frail Elderly (LIFE) pathway [13], and multidisciplinary care pathways [10, 11, 16, 18, 19].

There are contradictory findings in the literature regarding the effectiveness of the comprehensive geriatric care models. Some CGA pathways improved clinical outcomes such as decreased prevalence of delirium [17, 19], and LOS [10, 15–17], while other studies did not show any positive results [10, 13, 16]. The objective of this systematic review and meta-analysis is to determine the effects of geriatric care models in decreasing adverse outcomes versus standard care in the geriatric surgical patients. This systematic review and meta-analysis concentrates on the application of comprehensive geriatric care models in totality. It shows the importance of comprehensive geriatric care models in the current geriatric care. We hypothesize that there is an association between the geriatric care models and a decrease in the adverse outcomes such as prevalence of delirium, LOS, rates of 30-day admission, and mortality.

## Methods

We registered the protocol of this systematic review in the International Prospective Register of Systematic Reviews (PROSPERO) (registration number - CRD42020181779). The study was performed in accordance with the Preferred Reporting Items for Systematic Reviews and Meta-Analyses (PRISMA) guidelines [25].

Definition of CGA: Comprehensive geriatric assessment (CGA) is a multi-dimensional, multi-disciplinary process which consists of medical, mental, social and functional needs of the elderly people, and an integrated and coordinated care plan that includes treatment and long term follow up [26].

### Study selection criteria

Inclusion criteria were: 1) randomized and non-randomized controlled studies, prospective and retrospective cohort trials, that enrolled patients aged over 60 years, undergoing elective non-cardiac high-risk surgery; 2) must have CGA as a component of the geriatric care model; 3) must have an intervention group using geriatric care model and a control group (standard care); 4) reported at least one of the following postoperative outcomes in both the intervention and control group: prevalence of delirium, LOS, 30-day readmission rates, 30-day mortality, and any other postoperative complications; and 5) limited to the English language.

Exclusion Criteria were**:** 1) emergency surgical procedures and non-geriatric population studies. 2) ERAS program without a CGA component.

### Search strategy

We searched Medline, Pubmed, Embase, Cochrane Central Register of Controlled Trials, Cochrane Database of Systematic Reviews, Emcare Nursing, Web of Science, Scopus, CINAHL ClinicalTrials. Gov, and ICTRP (international Clinical Trials Registry Platform) for published and unpublished studies. The search strategy was developed with the help of information specialist (ME). We used both Medical Subject Headings (MeSH) and free text terms to identify relevant articles. Database searches were restricted from January 2009 to January 2020. The search strategy used controlled vocabulary terms and text word terms for each of the research topic components: care pathways and elderly and perioperative and study types. The full electronic search strategies used are shown in the Supplemental Digital Content (Appendix 1).

### Study process

The study authors prepared the pilot tested data collection form with the standard instruction for screening of the title, abstract, and full text, risk of bias assessment, data collection, and data analysis. Two reviewers (AS, SW) screened literature studies [27] (using Rayyan), assessed the risk of bias, collected data, and analysed independently. All conflicts were resolved by consensus and a third reviewer (FC).

### Risk of Bias assessment

For randomized controlled trials (RCT), we used a modified version of Cochrane Collaboration’s tool to assess the risk of bias [28]. The Cochrane tool; studies received a “low”, “high”, or “unclear” rating for each risk category. We considered random sequence generation; allocation concealment; blinding of outcome assessment; incomplete outcome bias; and selecting outcome reporting. The domain “blinding of participants and personnel” was removed from the quality assessment, as it was difficult to blind the CGA group (S-Table 1). For non-randomized studies, we evaluated study quality in accordance with the Meta-analysis Of Observational Studies in Epidemiology (MOOSE) guidelines [29] and Newcastle-Ottawa scale (NOS) [30]. Quality Assessment is included in S-Table 2. The key points of study quality reviewed included: i) a clear identification of the study population, ii) a clear definition of the outcomes and outcome assessment, iii) no selective loss of patients during follow-up, and iv) important confounders and/or prognostic factors identified. We evaluated each point using Yes/No. If one of these key points was not clearly mentioned in a study, it was considered a ‘No’. Each study was given a score using the Newcastle-Ottawa scale (S-Table 3).

### GRADE - quality of evidence

We assessed the quality of the evidence by Grading of Recommendations Assessment, Development, and Evaluation (GRADE). GRADE system includes the risk of bias, inconsistency, indirectness, inaccuracy, and publication bias. For each outcome, GRADE starts with a baseline rating of high (4 points) for RCT and low (2 points) for observational studies. The outcome rating then can be adjusted (downgraded) after considering the 5 assessment criteria (S-Table 4).

### Data extraction

Two reviewers (AS, SW) independently extracted the data using a standardised data collection form. The study characteristics for instance, author, year of publication, country of origin, study design, total sample size, sample size in intervention group, and control group were collected. Patient characteristics including age, gender, co-morbidities, surgical procedure, details about the intervention, and perioperative care/multidisciplinary teams were extracted.

Comprehensive geriatric care models which used CGA as the intervention were compared to control groups which received standard care with no intervention. We collected the primary and secondary outcomes from surgery to 30 days post-discharge from hospital. The primary outcomes reported were prevalence of delirium and LOS. The secondary outcomes were 30-days readmissions rates, 30-days mortality rates, and a number of other postoperative complications.

### Data analysis

We reported the results according to meta-analysis of observational studies in epidemiology (MOOSE) [29] and preferred reporting items for systematic reviews and meta-analyses (PRISMA) [25]. We pooled the data from RCTs and observational studies. Additionally, we explored the heterogeneity and pooled estimate based on the study type (RCTs vs. non-RCTs). The measure of association for postoperative outcomes was the weighted odds ratio (OR) with 95% confidence interval (CI) for dichotomous outcomes (delirium, readmission, mortality etc.), and the weighted mean difference (WMD) with 95% CI for the continuous outcome of LOS. The Mantel-Haenszel (M-H) method was used to combine dichotomous events, and the inverse variance method was used to combine continuous events. A high statistical heterogeneity was explored. In this analysis, the impact of each study on heterogeneity was explored by excluding studies one by one and recalculating the heterogeneity. We undertook sensitivity analyses for significant outcomes, by using alternative effect measures (odds ratio vs. risk ratio), pooling methods (Peto methods vs. Mantel-Haenszel method), and consideration on heterogeneity (random vs. fixed effect).

## Results

### Search results

**Fig. 1:**
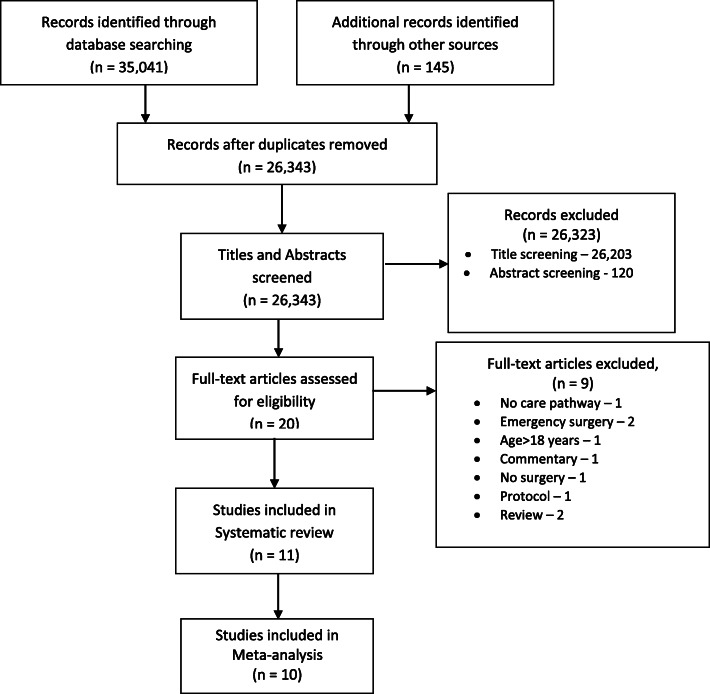
PRISMA study flow diagram

A complete search of the selected articles is summarized in Fig. 1, in accordance with the PRISMA statement [25]. A total of 35,186 articles was identified. After applying the deduplication process, 8843 articles were removed. The titles and abstracts of the remaining 26,343 articles were screened for selection criteria, after which 20 articles remained. Full-text screening of these 20 articles resulted in 11 articles which were included for the qualitative synthesis of the review [10, 11, 13, 15–19, 22–24]. We excluded nine articles due to no care pathway, no surgery, a non-geriatric population, or incorrect types of article.

### Patient and study characteristics

Eleven studies (RCTs: 4; non-RCTs: 7) included with 2672 patients (intervention group *n* = 1383, control group: *n* = 1289) [10, 11, 13, 15–19, 22–24]. An overview of the study and patient characteristics are summarized in Table 1. Most of the included studies are from Europe [Netherland (*n* = 3), United Kingdom (*n* = 1), Spain (*n* = 1), Sweden (*n* = 1) [13, 17–19, 23, 24], and United States (*n* = 4) [11, 15, 16, 31], while one is from China (*n* = 1) [10]. Patients in the included studies underwent cancer (*n* = 4) [13, 18, 19, 23], abdominal (*n* = 3) [10, 15, 16], vascular (*n* = 2) [11, 17], spinal (*n* = 1) [22], and total hip arthroplasty (*n* = 1) [24] surgeries. The included articles had the following study designs: RCT (*n* = 4) [10, 13, 17, 23], prospective cohort (*n* = 2) [11, 15], retrospective cohort (*n* = 3) [16, 19, 22], as well as pre-intervention and post-intervention study design (*n* = 2) [18, 24].

**Table 1:** Study and patient baseline characteristics

Author, Country & year	Study type	Type of Surgery	Sample Size (n)	Number of patients		Age (years)		Sex (Male)	
				Intervention	Control	Intervention	Control	Intervention-%	Control-%
Partridge [17], UK, 2017	RCT	Vascular	176	85	91	75 ± 6	75 ± 6	76.9	75.2
Hempenius, [13]Netherlands, 2013	RCT	Cancer	297	148	149	77 ± 6	77 ± 7	37.8	34.2
Hempenius, [23]Netherlands, 2016	RCT	Cancer	260	127	133	77 ± 6	77 ± 7	40.2	36.1
Chen [10], China, 2017	Cluster RCT	Abdominal	377	197	180	74 ± 5	74 ± 6	56.4	57.2
McDonald [15], USA, 2018	PC	Abdominal	326	183	143	75 ± 6	71 ± 6	51.0	46.6
Cronin [11] USA, 2011	PC	General or Vascular	69	26	43	75 ^a^	77 ^a^	46.2	23.3
Adogwa [22], USA, 2017	RC	Spine (lumber)	125	100	25	73 ± 6	73 ± 4	41.0	36.0
Tarazona-Santabalbina [19], Spain, 2019	RC	Colorectal Cancer	310	203	107	77 ± 4	75 ± 5	63.1	61.7
Nussbaum [16], USA, 2014	RC	Abdominal (pancreatico- duodenectomy)	242	100	142	65 ± 10	62 ± 11	39.0	47.0
Olsson [24], Sweden, 2014	Pre-post	THA	266	128	138	68 ± 12	66 ± 13	35.6	35.5
Souwer [18], Netherlands, 2018	Pre-post	Colorectal cancer (Laparoscopic surgery)	224	86	C1–63	80.6 (6.2)	C1–81.4 (7.3)	42.0	C1–52.0
					C2–75		C2–79.7(5)		C2–51.0

*Abbreviations*: *C1* Control1 (2010–2011); *C2* Control2 (2012–2013), *PC* Prospective cohort; Pre-post, Pre-intervention Post-intervention study design, *RC* Retrospective cohort, *RCT* Randomized Controlled Trial, *THA* Total Hip Arthroplasty. Data expressed as Mean ± SD, median (IQR) unless otherwise stated, *IQR* Interquartile range. ^a^represent mean years

### Study quality assessment – risk of bias

Using the Cochrane tool, the four RCT’s had low bias on most of the domains. Random sequence generation and incomplete outcome data were the most sufficiently addressed, with all four studies reporting low bias in these domains. Allocation concealment and blinding of outcome assessment were the least sufficiently addressed. In allocation concealment domain, two studies reported low bias and two reported high bias. Similarly, two studies reported low bias and two reported unclear bias in outcome assessment domain [32]. (S-Table 1) According to the Newcastle Ottawa scale scoring system, the quality of the six non-RCTs ranked from 7 to 9 indicated low risk of bias [30]. One study was considered being of high risk due to selection and outcome bias [18]. (S-Tables 2 and 3).

### GRADE evaluation

GRADE evaluation of the quality of evidence for the outcomes: prevalence of delirium, LOS, 30-days readmission, and 30-days mortality was conducted. The quality of evidence of the RCTs and non-RCTs together on delirium, LOS, 30-days readmission, and 30-days mortality was rated very low due to risk of bias and imprecision, respectively. (S-Table 4).

### The different comprehensive geriatric care models

Overview of the different comprehensive geriatric care models is summarized in Table 2. All geriatric care models contained CGA, which is an established multi-domain assessment addressing patients’ physiological, social, psychological, and functional state before surgery [17]. The primary feature is cognitive status screening and intervention protocol directed to cognitive impairment. Proactive care of Older People (POPS) [17] model referred patients to specialists following diagnosis of cognitive impairment or delirium, while the majority followed recommendations from caring physicians based on the initial cognitive assessment [11, 13, 15, 19, 22–24]. Some studies did not have cognitive impairment intervention [16, 24], while others have devised models for addressing impairment, such as orientation communication in the Hospital Elder Life Program (HELP) [10, 14]. Intervention tools to specifically address frailty/functional status were included in all the models, with one exception [16]. Intervention tools either took the form of exercise regimens [10, 14, 19], or tailored plan determined after assessment [13, 15, 17, 22, 23], while one study referred to general rehabilitation efforts [18].

**Table 2:** Comprehensive geriatric care models

Geriatric care model	POPS (Harari et al) [31]	POSH (McDonald et al) [15]	HELP (Inouye et al) [14]	gPCC (Ekman et al) [12]	LIFE (Hempenius et al) [13]	MDC^a^
Author, study type	Partridge,[17]RCT	McDonald [15], PC Adogwa [22], RC	Chen [10], Cluster RCT	Olsson [24], Pre-post	Hempenius [13], 2013, RCT Hempenius [23], 2016, RCT	Cronin [11], PC Tarazona-Santabalbina [19], RC Nussbaum, [16]RC Souwer [18], Pre-post
Pre-operative	1. CGA 2. Assessment of Cognitive Function, Frailty, Anaemia, Cardiac evaluation	1. CGA 2. Risk assessment focused on -• Cognition• Mobility • Functional status • Co-morbidities Medications • Nutrition • Hydration • Pain • Advanced care planning	1. CGA 2. Screened for 6 delirium risk factors: • Cognitive impairment • Immobility • Sleep deprivation • Dehydration • Vision impairment • Hearing impairment	1. CGA includes: • Need for additional support after discharge • ADL level • Social lifestyle • Symptoms severity 2.Patient - provider joint Rx plan	1. CGA 2. Checklist to standardize intervention • Mobility • Co-morbidities • Nutrition • Loss of vision & hearing •Medication • Depression •Incontinence • Cognitive, social & instrumental ADL • Delirium ICP	1. CGA [18, 19] 2. Rehab care included training, dietary, cognitive, & emotional guidance [18] 3. Nutritional assessment [19] 4. Risk assessment for functional (VES) & polypharmacy status [11]
Post-operative	• CGA • ICP • Home visit follow-up therapy	• Mx of co-morbidity & pain • Delirium assessment • Enhancement of mobility & nutrition • Counselling for discharge & post-hospital care assessment	• Orientation • Therapeutic activities • Early mobilization • Feeding assistance • Sleep enhancement • Vision & hearing reinforcement •Delirium	• Shared decision: Patient-provider partnership •Documentation: Decisions & assessments according to PCC	• Geriatric nurse daily visit	• Follow-up using postoperative order set assessing functionality, pain & medication [11] •Nutritional assessment & FTRP [16]
Polypharmacy	NR	Reduction recorded	NR	NR	NR	Recorded [18]
Delivery team	•Geriatrician • Nurse specialist •Occupational therapist	• Geriatrician • Nurse • Surgeons •Anaesthesiologists	• Geriatrician • Geriatric nurse • Pharmacist • Nutritionist • Rehab therapists • Trained volunteers	• Physicians • Surgeons • Nurse •Physiotherapists •Occupational therapists • Patient representatives	•Geriatrician • Geriatric nurse	•Geriatrician • Geriatric nurse • Oncology nurse • Surgeons • Residents • Dieticians • Physical therapists

*Abbreviations*: *ADL* Activities of daily living, *CGA* Comprehensive geriatric assessment, *FTRP* Fast-track recovery pathway, *gPCC* Gothenburg person centred care, *HELP* Hospital Elder Life Program, *ICP* Individual care plan, *LIFE* Liaison Intervention in Frail Elderly, *MDC* Multidisciplinary care, *NR* Not recorded, *PC* Prospective cohort, *POPS* Proactive care of older people undergoing surgery, *POSH* Perioperative Optimization of Senior Health, *Rehab* Rehabilitation, *RC* Retrospective cohort, *RCT* Randomized controlled trial, *Rx* Treatment, *Pre-post* Pre-intervention and post-intervention design, *VES* Vulnerable elder survey

^a^The pathways which were not using standard care models (like HELP, POPS, POSH, etc.) were grouped into the MDC group

### Post-operative outcomes

#### Delirium

**Fig. 2:**
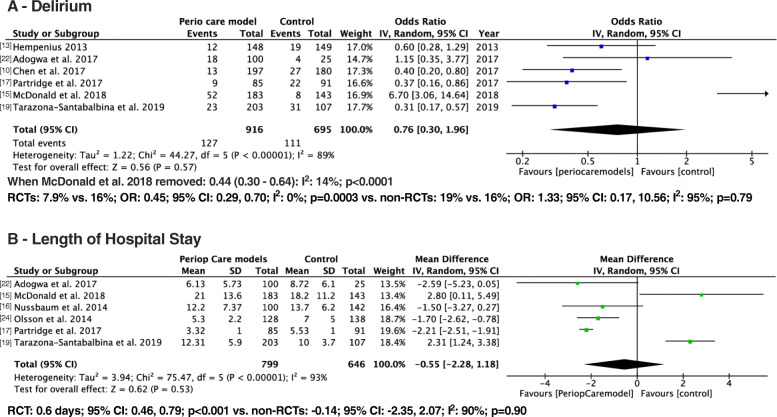
Forest plot displaying a meta-analysis of the delirium and LOS

Six studies consisting of 916 patients in the intervention group and 695 patients in the control group reported the prevalence of delirium. Even though the prevalence of delirium was 2.1% less in the intervention group compared to the control group, it was not significant statistically (13.8% vs 15.9%; OR: 0.76; 95% CI: 0.30–1.96; I^2^: 89%; *p* = 0.57) (Fig. 2a). Our influential analysis showed that McDonald et al. 2018 contributed the maximum heterogeneity. When this study was removed and pooled prevalence of delirium was recalculated, the heterogeneity decreased by 75%, and the prevalence of delirium was significantly less in the intervention group compared to control group (10.2% vs. 18.6%; OR: 0.44; 95% CI: 0.30–0.64; I^2^: 14%; *p* < 0.0001). We conducted the subgroup analysis based on the type of study (RCTs vs. non-RCTs). The prevalence of delirium was reported in three RCTs [10, 13, 17], and three non-RCTs [15, 19, 22]. .Among the RCTs [10, 13, 17], the prevalence of delirium was significantly less in the intervention (*n* = 430) compared to the control group (*n* = 420) (7.9% vs. 16%; OR: 0.45; 95% CI: 0.29, 0.70; I^2^: 0%; *p* = 0.0003). The absolute risk reduction for the prevalence of delirium is 8.28% (95% CI: 3.9, 12.6), and the number needed to treat is 13 (95% CI: 7.9, 25). The pooled estimate remained significant, and heterogeneity remained at zero after conducting the sensitivity analysis for this significant outcome. Among the non-RCTs [15, 19, 22], there was no significant difference between the two groups on the prevalence of delirium (intervention (*n* = 486) vs. control: (*n* = 275) (19% vs. 16%; OR: 1.33; 95% CI: 0.17, 10.56; I^2^: 95%; *p* = 0.79).

#### Hospital length of stay (LOS)

Eight studies reported data on LOS, three from RCTs [10, 13, 17], and five from non-RCTs [15, 16, 19, 22, 24]. However, two RCTs were excluded from meta-analysis due to clinical heterogeneity as a lot of patients stayed in ICU postoperatively [10, 13].

In the pooled estimate from six studies (five non-RCTs and one RCT) with different care models, there was no significant difference in the LOS between the intervention (*n* = 799) and control groups (*n* = 646) (mean difference: -0.55; 95% CI: − 2.28, 1.18; I^2^: 93%; *p* = 0.53) [15–17, 19, 22, 24] (Fig. 2b).

#### 30*-*days re-admissions

**Fig. 3:**
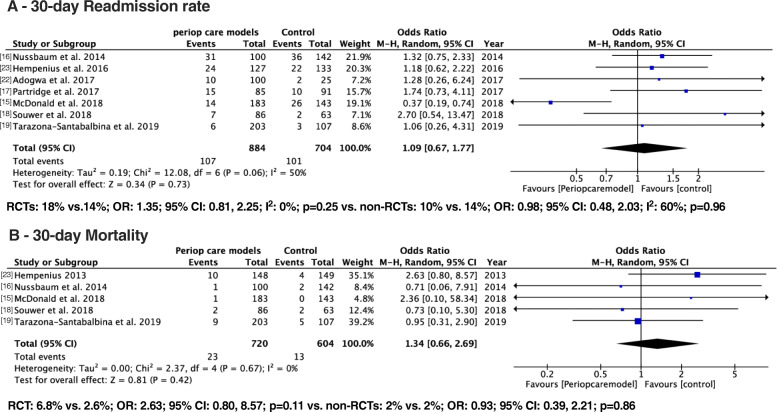
Forest plot displaying a meta-analysis of 30-days readmission and 30-mortality. Abbreviations: RCT, Randomized Controlled Trials; Non-RCT, Non- Randomized Controlled Trials; LOS, Length of hospital stay

Seven studies consisting of 884 patients in the intervention group and 704 patients in the control group reported on 30-days re-admission. Overall, there was no significant difference in the 30-day readmission rates. The 30-day re-admission rates were 12.1% in the intervention group compared to 14.3% in the control group (OR: 1.09; 95% CI: 0.67–1.77; I^2^: 50%; *p* = 0.73) (Fig. 3a). Subgroup exploration based on the type of study (RCTs vs. non-RCTs) did not show any significant difference in the 30-day readmission rates. Out of seven studies, two RCTs measured the rate of the 30-day readmission rates (intervention vs. control: 18% vs.14%; OR: 1.35; 95% CI: 0.81, 2.25; I^2^: 0%; *p* = 0.25) [17, 23] and five non-RCT studies provided data on the 30-day readmission rates (intervention vs. control: 10% vs. 14%; OR: 0.98; 95% CI: 0.48, 2.03; I^2^: 60%; *p* = 0.96) [15, 16, 18, 19, 22].

#### 30-days mortality

Five studies consisting of 720 patients in the intervention group and 604 patients in the control groups reported on 30-day mortality. The pooled data on 30-day mortality was not significantly different between the intervention vs. control group (3.2% vs. 2.1%; OR: 1.34; 95% CI: 0.66–2.69; I^2^: 0%; *p* = 0.42). Out of five studies, only one RCT reported data on 30-day mortality (6.8% vs. 2.6%; OR: 2.63; 95% CI: 0.80, 8.57; *p* = 0.11) [13] and the pooled data from the four non-RCTs did not show significantly difference between the intervention vs. control group (2% vs. 2%; OR: 0.93; 95% CI: 0.39, 2.21; *p* = 0.86) [15, 16, 18, 19] (Fig. 3b).

#### Other postoperative outcomes

S-Table 5 contains detailed secondary outcomes. Complications such as pneumonia (*n* = 3) [15, 17, 22], discharge to home with self-care (*n* = 2) [15, 17], activities of daily living (ADL) (*n* = 2) [23], functional status at 30 days [11], and geriatric syndromes, and events [19] were reported in some studies. Three studies reported no significant difference in pneumonia [15, 17, 22]. The percentage of patients discharged to home with self-care was reported in two studies [15, 17], with one study reporting a significant difference of *p* = 0.04 [15]. The study reporting ability to achieve ADL did not show any improvement, but functional status at 30 days was significantly improved (*p* < 0.01). Similarly, geriatric syndromes and events improved significantly with a *p*-value < 0.001 [19].

## Discussion

To our knowledge, there was no previous systematic review or meta-analysis on comprehensive geriatric care models and their effects on postoperative outcomes in elderly population undergoing surgery. Our systematic review and meta-analysis yielded eleven studies on geriatric care models with CGA as the major component. We found these care models did not show a significant difference in the prevalence of delirium, LOS, 30-days readmission rates, and 30-day mortality.

### Characteristics of comprehensive geriatric care model

Of the eleven studies [10, 11, 13, 15–19, 22–24] most adapted six comprehensive geriatric care models [11–16, 18, 19, 33]. This systematic review allowed for a comparison of the similarities and differences in the different care models. All geriatric care models used CGA. CGA identifies the co-morbid conditions of elderly patients, thus helping health care professionals optimize perioperative care. It allows the opportunity for counselling patients regarding risk reduction and evaluation of nonsurgical treatment options [34]. Most of the programs centred around management of symptoms of cognitive impairment, frailty, and immobility, showcasing the importance of managing impairment before surgery as a preventative measure and after surgery to improve postoperative adverse outcomes.

ERAS pathways outline preoperative and postoperative management, supplemented by intraoperative risk reduction efforts such as specific administration of analgesics, nausea and vomiting, and hemodynamic management [35, 36], The American Society for Enhanced Recovery and Perioperative Quality Initiative and the European Society of Anaesthesia have echoed the importance of assessing preoperative delirium risk and informing patients of their status before surgery [34, 35]. Thus, both ERAS and geriatric care models use delirium assessment and management. Our review excluded ERAS pathways because they differ from geriatric care models which put a primary emphasis on CGA. Also, the involvement of the geriatric team for CGA is important besides a multidisciplinary team of surgeons, anaesthesiologists, pharmacists, occupational therapists/physiotherapists, and nutritionists. The multidisciplinary team approach has been credited for the disciplined implementation of the care model [18].

### Outcome

Although the prevalence of delirium was 2.1% less in the intervention group vs the control group in six studies, it was not significant statistically. However, in the three RCTs, the prevalence of delirium was reduced by over 50% in the intervention group, with an absolute risk reduction of 8% and Number Needed to Treat (NNT) of 13. The high prevalence of delirium and its correlation with diminished quality of life and cognitive impairment emphasizes the importance of selecting it as a clinical outcome. Both the Hospital Elder Life Program (HELP) [14], and Proactive care of Older People (POPS) [17], care models found significant reduction in the prevalence of delirium. Both referred patients to specialists following CGA and offered a comprehensive care model towards improvement of delirium [10, 17]. On the contrary, the other models provided only recommendations for recovery after assessment [13, 15, 19, 22]. This may have accounted for the differences in the prevalence of delirium in these care models.

The LOS is a common clinical outcome to evaluate the effectiveness of interventions [23]. A shortened LOS leads to decreased cost and reduction in the probability of contracting infections [37, 38]. Although variation in measurement of outcomes limited the ability of pooled estimate among the RCTs, all the programs showed a reduction in LOS except of one using the Liaison Intervention in Frail Elderly (LIFE) model [13].

Readmission to hospital has gained popularity as an outcome measure in quality improvement. Literature is divided over its validity due to the inconsistent correlation between hospital readmission and other clinical outcomes [39]. Our meta-analysis results showed no significant differences in 30-day readmission between the intervention and control groups. The only geriatric care model showed significant result was the Perioperative Optimization of Senior Health (POSH) model [15]. Although our review showed that CGA only decreases the prevalence of delirium by over 50% in elderly patients, Eamer et al. [40] found CGA decreased mortality in hip fracture patients, and Ellis et al. [26] showed the CGA improved patients being able to live at home.

We rated the quality of the evidence for outcomes using GRADE system [41]. The ratings were very low for all the outcomes when we combined RCTs and non-RCTs, (indicating considerable uncertainty regarding the estimates of effect**).** In our research, we found that there was no significant difference among intervention and control groups. However, further RCTs of adequate power and clearly defined endpoints in specific surgical procedures are warranted to determine the overall benefits in the postoperative outcomes.

### Limitations

There are some limitations in this systematic review and meta-analysis. There are only four RCTs with 7 non-RCTs, and the studies were heterogeneous with different types of surgeries. Eight studies were single centre studies [10, 11, 15–17, 19, 22, 24] with three multi-centre studies [13, 18, 23]. The chance of bias increases as the staff between the intervention and control groups is the same augmenting the chance of contamination. Also, including pre-post design interventions introduces bias due to the lack of randomization. Furthermore, the multidisciplinary approach varied from between studies as they were conducted in different institutions and at different time periods. This may cause lack of uniformity in the intervention and may have contributed to another source of bias. The studies included were in English and we may have missed studies in other languages. Nevertheless, this systematic review provides a summary of existing evidence and serves as an impetus for the academic community to do further research in this area.

## Conclusion

The comprehensive geriatric care models involved pre-operative CGA, and intervention tools to address cognition, frailty, and functional status. In non-cardiac high-risk surgeries, geriatric care models with CGA, there was no cumulative significant differences in the prevalence of delirium, LOS, 30-days readmission rates, and 30-day mortality. Further RCTs are needed to delineate the benefits of comprehensive geriatric care model on postoperative outcomes.

## Supplementary Information

**Additional file 1: Supplementary Table 1.** Cochrane Risk of Bias assessment for RCTs. **Supplementary Table 2.** Study quality assessment and risk of bias for non-RCTs. **Supplementary Table 3.** Newcastle-Ottawa scale (NOS) for non-RCTs. **Supplementary Table 4.** GRADE evaluation of evidence quality. **Supplementary Table 5.** Summary of postoperative outcome results. File format (three-letter file extension) -PDF (Adobe Acrobat) (.pdf). Data description - Risk of bias assessment, quality assessment, NOS, GRADE analysis, and postoperative outcome results of delirium, LOS, 30-days readmission rate and 30-days mortality. (All these files are attached in one PDF).

**Additional file 2.** PRISMA checklist.

**Additional file 3.** Search strategy.

## Data Availability

All data generated or analysed during this study are included in this article [and its supplementary information files].

## Abbreviations

CGA: Comprehensive geriatric assessment
LOS: Length of hospital stay
RCT: Randomized controlled trial
PC: Prospective cohort
RC: Retrospective cohort
Pre-post: Pre-intervention and post-intervention study design
ERAS: Enhanced recovery after surgery
Intervention group: Comprehensive geriatric care model with CGA as a chief component
