# Supplementary material for: Effects of comprehensive geriatric care models on postoperative outcomes in geriatric surgical patients: a systematic review and meta-analysis
Source: BMC Anesthesiol. 2021 Apr 22;21:127. doi: 10.1186/s12871-021-01337-2 (PMC8061210; doi:10.1186/s12871-021-01337-2)
Supplement: Supplementary file 1 — Additional file 1: Supplementary Table 1. Cochrane Risk of Bias assessment for RCTs. Supplementary Table 2. Study quality assessment and risk of bias for non-RCTs. Supplementary Table 3. Newcastle-Ottawa scale (NOS) for non-RCTs. Supplementary Table 4. GRADE evaluation of evidence quality. Supplementary Table 5. Summary of postoperative outcome results. File format (three-letter file extension) -PDF (Adobe Acrobat) (.pdf). Data description - Risk of bias assessment, quality assessment, NOS, GRADE analysis, and postoperative outcome results of delirium, LOS, 30-days readmission rate and 30-days mortality. (All these files are attached in one PDF). [file 12871_2021_1337_MOESM1_ESM.pdf]

Supplementary Table 1- Cochrane Risk of Bias assessment (RoB1)

1a Summary

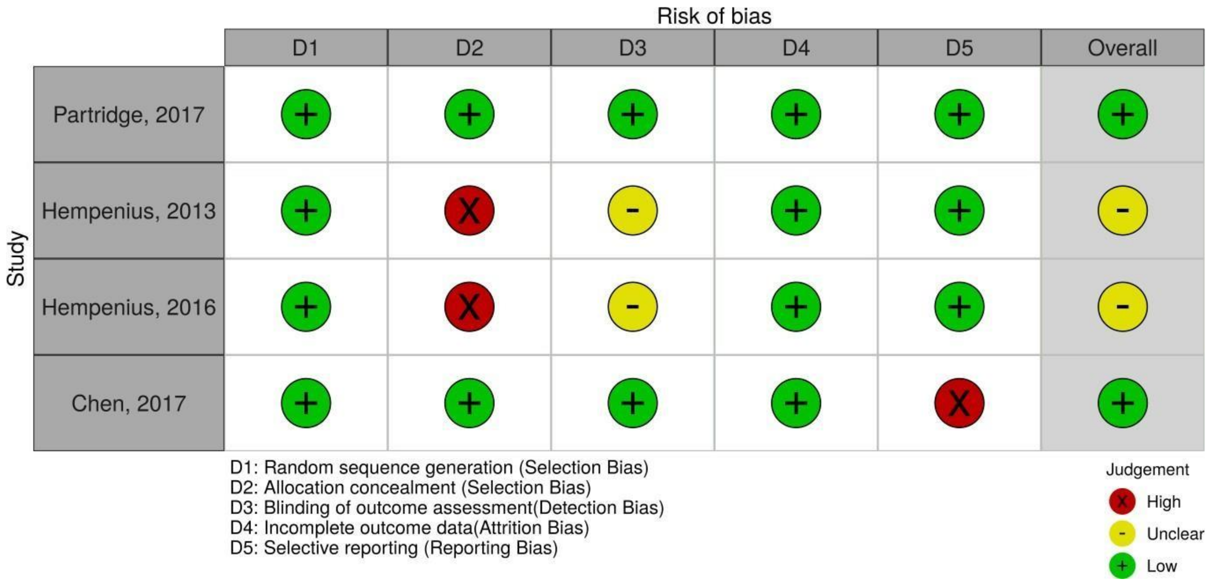

1b Weight plot

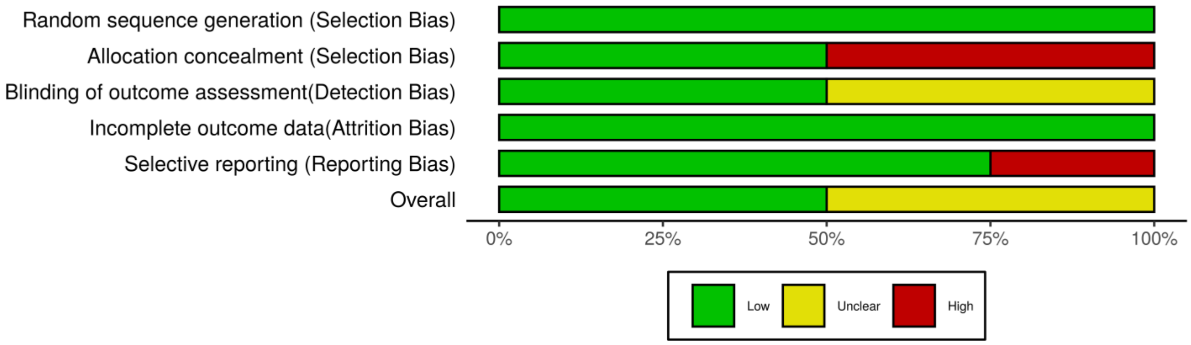

Supplementary Table 2 – Study quality assessment and risk of bias

Study ID	Study design	Study population clearly identified?	Clear definition of outcome and outcome assessment?	Selective loss of patients during the follow up?	Important confounders and / or prognostic factors identified	Newcastle-Ottawa scale scores
McDonald ,[15] 2018	PC	Yes	Yes	No	Case-matched control group. Confounders: age, number of comorbid conditions, laparoscopic vs. open, ERAS. Multivariate regression model applied both alone and in combination. Logistic regression for dichotomous and ordinary least squares regression for continuous outcomes.	9
Cronin ,[11] 2011	PC	Yes	Yes	No	Baseline characteristics compared, but confounders not identified. No regression analysis done.	7
Adogwa,[22] 2017	RC	Yes	Yes	No	Baseline characteristics compared, but confounders not identified. No regression analysis done.	7
Tarazona-Santabalbina,[19] 2019	RC	Yes	Yes	No	A stepwise binary logistic regression was used to create multivariate model. Logistic regression done.	7
Nussbaum ,[16]2014	RC	Yes	Yes	No	Univariate and multivariate analysis done by calculating the inverse logarithm of the beta coefficient	8
Olsson ,[24] 2014	Pre-post	No	Yes	No	Baseline characteristics compared, comorbid conditions, Fisher's exact test for dichotomous variables, the Mantel–Haenszel Chi-squared test for ordered categorical variables.	8
Souwer,[18]2018	Pre-post	No	Yes	No	Patient characteristics campared, age, Short Nutritional Assessment Questionnaire, prehabilitation and rehabilitation, ANOVA, logistic regression	5

Supplementary Table 3: Newcastle-Ottawa scale[29]

Quality assessment criteria	Acceptable	McDonald ,[15] 2018	Cronin, [11]2011	Adogwa, [22]2017	Tarazona-Santabalbina,[19] 2019	Nussbaum, [16] 2014	Olsson, [24] 2014	Souwer, [18] 2018
**Selection**								
Representativeness of exposed cohort?	Representative of average adult in Preoperative period	*	*	*	*	*	*	-
Selection of the non-exposed cohort?	Drawn from same community as exposed cohort	*	*	*	*	*	*	-
Ascertainment of exposure?	Secured records, Structured interview, questionnaire	*	*	*	*	*	*	*
Demonstration that outcome of interest was not present at start of study?		*	*	-	-	-	*	-
**Comparability**								
Study controls for age/sex?	Yes	*	*	*	*	*	*	*
Study controls for at least 3 additional risk factors?	Age, Gender, Co-morbidity etc	*	-	-	*	*	*	*
**Outcomes**								
Assessment of Outcome?	Independent blind assessment, record linkage	*	-	*	-	*	-	-
Was follow-up long enough for outcome to occur?	Follow-up	*	*	*	*	*	*	*
Adequacy of follow-up of cohorts?	Complete follow-up, or subjects lost to follow-up unlikely to introduce bias	*	*	*	*	*	*	*
**Overall Quality Score (Maximum = 9)**		9	7	7	7	8	8	5

Supplementary Table 4: GRADE evaluation of evidence quality

CGA compared to standard care for geriatric patients undergoing high risk surgery.

Patient or population: Geriatric patients undergoing high risk surgery
Setting: Hospitals

Outcomes	No. of participants (studies)	Certainty of the evidence (GRADE)	Relative effect (95% CI)	Anticipated absolute effects	
				Risk with [Standard care]	Risk difference with [CGA]
In-Hospital Length of stay (RCTs + Non-RCTs)	1445 (6 observational studies) *	⊕◯◯◯ VERY LOW [a,b]	-	The mean in-Hospital Length of stay (RCTs + Non-RCTs) was **0**	MD **0.55 lower** (2.28 lower to 1.18 higher)
Delirium (RCTs + Non-RCTs)	1611 (6 observational studies)	⊕◯◯◯ VERY LOW [c,d]	**OR 0.76** (0.30 to 1.96)	160 per 1,000	**33 fewer per 1,000** (106 fewer to 112 more)
30-day Readmission Rates (RCTs + Non-RCTs)	1588 (7 observational studies)	⊕◯◯◯ VERY LOW [e]	**OR 1.09** (0.67 to 1.77)	143 per 1,000	**11 more per 1,000** (43 fewer to 85 more)
30-day Mortality (RCTs + Non-RCTs)	1324 (5 observational studies)	⊕◯◯◯ VERY LOW [e,f]	**OR 1.34** (0.66 to 2.69)	22 per 1,000	**7 more per 1,000** (7 fewer to 34 more)

The risk in the intervention group (and its 95% confidence interval) is based on the assumed risk in the comparison group and the **relative effect** of the intervention (and its 95% CI).

CI: Confidence interval; **MD:** Mean difference; **OR:** Odds ratio

CGA compared to standard care for geriatric patients undergoing high risk surgery.

Patient or population: Geriatric patients undergoing high risk surgery
Setting: hospitals

Outcomes	№ of participants (studies) Follow up	Certainty of the evidence (GRADE)	Relative effect (95% CI)	Anticipated absolute effects	
				Risk with [Standard care]	Risk difference with [CGA]

GRADE Working Group grades of evidence
High certainty: We are very confident that the true effect lies close to that of the estimate of the effect
Moderate certainty: We are moderately confident in the effect estimate: The true effect is likely to be close to the estimate of the effect, but there is a possibility that it is substantially different
Low certainty: Our confidence in the effect estimate is limited: The true effect may be substantially different from the estimate of the effect
Very low certainty: We have very little confidence in the effect estimate: The true effect is likely to be substantially different from the estimate of effect

Explanations

* We downgraded the level of evidence as most of the studies were observational studies with small sample size.
a. Outcome assessment and selection bias
b. Heterogeneity is(I^2) is 93%. after doing sensitivity analysis also I^2 did not change considerably.
c. Allocation concealment (Selection bias) in Hempenius,2013, and selective reporting in Chen, 2017 study are at high risk.
d. Heterogeneity is (I^2) is 89%
e. The domain allocation concealment and selection bias are at a high risk.
f. CI is very wide 0.66 to 2.69

Supplementary Table 5: Summary of postoperative outcome results

Table 5A: Delirium prevalence and LOS (Length of stay)

Authors, year	Intervention	Control	p-value
**Delirium prevalence– n(%)**			
McDonald,2018[15]	52(28.4)	8(5.6)	<0.001
Partridge,2017[17]	9(11)	22(24)	0.018
Tarazona-Santabalbina, 2019[19]	23(11.3)	31(29.2)	<0.001
Chen,2017[10]	13(6.6)	27(15.1)	0.008
Hempenius, 2016[23]	26/227(11.5) *		NS
Hempenius,2013[13]	12(9.4)	19(14.3)	NS
Adogwa, 2017[22]	18(18)	4(16)	0.81
**LOS (days)**			
McDonald ,2018[15]	21 ± 13.6	18.2 ± 11.2	<0.001
Partridge, 21017[17]	3.32 ± 1	5.53 ± 1	<0.001
Tarazona-Santabalbina, 2019[19]	12.31 ± 5.9	10 ± 3.7	0.208
Chen, 2017[10]	6.3 ± 3.73	8 ± 5.9	0.04
Hempenius, 2013[13]	63(49.6)	57(42.9)	NS
Olsson, 2014[24]	5.3 ± 2.2	7 ± 5.0	<0.0005
Souwer, 2018[18]	5(6)	C1- 17(27)<br>C2- 10(13)	(C1-I)0.047<br>(C2-I) 0.001
Nussbaum, 2014[16]	12.2 ± 7.37	13.7 ± 6.2	0.015
Adogwa, 2017[22]	6.13 ± 5.73	8.72 ± 6.10	0.06

Data expressed as Mean±SD, n(%) unless otherwise stated.
C1- Control 1(2010-2011), C2- Control 2(2012-2013).
* Postoperative delirium occurred in 26 out of 227 patients (11.5%).

Table 5B: 30 days readmission rate and 30 days mortality

Authors, year	Intervention	Control	p-value
**30 days readmission rate – n(%)**			
McDonald, 2018[15]	14(7.8)	26(18.3)	0.004
Partridge, 2017[17]	15(18)	10(11)	0.193
Tarazona-Santabalbina, 2019[19]	6(3)	3 (2.8)	1
Nussbaum, 2014[16]	31(31)	36(25.4)	0.85
Souwer, 2018[18]	7(8)	C1- 2(35)<br>C2- 6(8)	NS
Adogwa, 2017[22]	10(10)	2(8)	0.77
**30 days mortality – n(%)**			
Tarazona-Santabalbina, 2019[19]	9(4.4)	5(4.7)	1
Hempenius, 2016[23]	17(13.4)	9(6.8)	NS
Hempenius, 2013[13]	10(7.9)	4(3)	NS
Adogwa,2017[22]	0(0)	0(0)	0.99
Nussbaum,2015[16]	1(1)	2(1.4)	>0.999
Souwer, 2018[18]	2(2)	C1- 2(3)<br>C2-1(1)	NS

Table 5C: Other postoperative outcomes

Authors, year	Intervention	Control	p-value
**No. of complications – n(%)**			
Hempenius, 2013[13]	42(33.1)	38(28.6)	NS
Nussbaum, 2014[16]	43(43)	53(41)	0.792
McDonald, 2018[15]	82(44.8)	84.58.7)	<0.001
Souwer, 2018[18]	25.8(30)	C1- 23.9(38)<br>C2- 21.8(29)	NS
Tarazona-Santabalbina, 2019[19]- (Mean ± SD)	3±2.2	2.4±2.7	0.069
**Pneumonia – n(%)**			
McDonald, 2018[15]	3(1.6)	2(1.4)	>0.99
Partridge, 2017[17]	8(9)	12(13)	0.43
Adogwa, 2017[22]	5(5)	1(4)	0.82
**Discharge home with self care – n(%)**			
McDonald, 2018[15]	114(62.3)	73(51.1)	0.04
Partridge, 2017[17]	4(4.7)	12(13.18)	0.51
**Pain level (Day 2)**			
Cronin, 2011[11]	2.06	3.29	0.09
Pain level is on scale of 0 (no pain) to 10 (maximum pain)			
**Functional status at 30 days (VES score)-Vulnerable elder Survey**			
Cronin, 2011[11]	0.45	2.28	<0.01
**ADL functioning –n(%)**			
Hempenius, 2016[23]	64(60.4)	68(56.2)	NS
**Geriatric syndromes and events -n (%)**			
Tarazona-Santabalbina, 2019[19]	21 (10.3)	28 (26.2)	<0.001
