# Supplementary material for: Effects of comprehensive geriatric care models on postoperative outcomes in geriatric surgical patients: a systematic review and meta-analysis
Source: BMC Anesthesiol. 2021 Apr 22;21:127. doi: 10.1186/s12871-021-01337-2 (PMC8061210; doi:10.1186/s12871-021-01337-2)
Supplement: Supplementary file 3 — Additional file 3. Search strategy. [file 12871_2021_1337_MOESM3_ESM.pdf]

Supplementary Digital Content (Appendix 1): Search strategy

Attached is the search for:

1. Systematic review searching for: Perioperative Care Pathway and Elderly and Surgery and Studies; limited to human, elderly (>65 years of age), English language; no conference literature.

The databases searched were: (by ME)

1. Medline; 2. Medline In-Process/ePubs; 3. Embase; 4. CCTR; 5. CDSR; 6.Ovid Emcare Nursing; 7. CINAHL; 8. Web of Science; 9. Scopus; 10. ClinicalTrials.Gov; 11. WHO ICTRP.

RESULTS & STRATEGY USED: *see following*

Search strategy saved as: 2020-01-21 FC Perioperative Care Pathway and Elderly and Surgery and Studies - Searches

Contents

Medline 2
Medline In-Process 11
Embase 21
CCTR 28
CDSR 34
Ovid Emcare Nursing 38
CINAHL 46
Web of Science 50
Scopus 52
ClinicalTrials.Gov 54
WHO ICTRP 54

Medline

Ovid MEDLINE(R) 1946 to January 22, 2020

#	Searches	Results
1	Algorithms/ and ("Patient Care"/ or "Progressive Patient Care"/ or "Patient Care Planning"/ or "Patient Care Management"/ or "Episode of Care"/)	650
2	Clinical Protocols/	27377
3	"Continuity of Patient Care"/	18698
4	Critical Pathways/	6555
5	Decision Support Techniques/	19758
6	Decision Theory/	923
7	exp Benchmarking/	12998
8	exp Decision Trees/	10874
9	exp Guideline/	32640
10	exp Guidelines As Topic/	156836
11	exp Practice Guideline/	25758
12	exp Practice Guidelines As Topic/	114749
13	Guideline Adherence/	31214
14	Health planning guidelines/	4071
15	Models, Organizational/	18746
16	Patient Care Bundles/	753
17	Physician's Practice Patterns/	57876
18	algorhythm*.mp.	114

19	algorism*.mp.	57
20	(algorithm* and care).mp.	23673
21	bench mark*.mp.	285
22	benchmark*.mp.	34816
23	decision tree?.mp.	14546
24	flow chart?.mp.	1255
25	flow diagram???.mp.	499
26	flowchart?.mp.	975
27	((comply or complies or compliant or compliance) adj2 (policy or policies)).mp.	233
28	((comply or complies or compliant or compliance) adj2 protocol*).mp.	694
29	(best adj2 practi#e?).mp.	21378
30	(care adj (bundle or bundles)).mp.	1118
31	"care intervention?".mp.	4396
32	(care adj2 (path or paths or pathway or pathways)).mp.	4267
33	(care adj2 map*).mp.	371
34	care model?.mp.	5801
35	(care adj2 plan*).mp.	53208
36	(clinical adj1 (path or paths or pathway or pathways)).mp.	3218
37	(clinical adj2 protocol?).mp.	34918
38	(comprehensive adj2 care).mp.	12948
39	(coordinated adj2 care).mp.	1507
40	(co-ordinated adj2 care).mp.	80
41	(critical adj2 (path or paths or pathway or pathways)).mp.	10927
42	(decision adj2 tree?).mp.	14624
43	(decision? adj2 aid?).mp.	4355
44	(decision? adj2 analy*).mp.	9397
45	(decision? adj2 model*).mp.	7961
46	(decision? adj2 techni*).mp.	20481
47	((framework? or frame work?) and care).mp.	38170
48	((guideline or guidelines) adj2 care).mp.	3633
49	(guidance adj2 (introduc* or issu* or impact* or effect* or disseminat* or distribut* or implement*)).mp.	1551
50	(management adj2 protocol*).mp.	3975
51	(multicomponent adj2 (path? or pathway?)).mp.	28
52	(multi-component adj2 (path? or pathway?)).mp.	12
53	(multicomponent adj2 (program? or programme?)).mp.	301
54	(multi-component adj2 (program? or programme?)).mp.	120
55	(multimodal adj2 (path? or pathway?)).mp.	74
56	(multimodal adj2 (program? or programme?)).mp.	473
57	(multi-modal adj2 (program? or programme?)).mp.	75
58	(nurs* adj2 protocol*).mp.	647
59	(optimal* adj2 care).mp.	5605

60	(optimi* adj2 care).mp.	3265
61	optimi#ation.mp.	99097
62	(organi#ational adj1 model?).mp.	19268
63	((policy or policies) adj2 care).tw.	5268
64	(practi#e adj1 parameter?).mp.	1043
65	(practi#e adj1 pattern?).tw.	6840
66	(practi#e adj2 (protocol* or policy or policies or guideline*)).mp.	156024
67	(proactive* adj2 care).mp.	347
68	(process?? adj2 (chart? or diagram* or flowchart*)).mp.	405
69	((program or programme) adj project?).mp.	254
70	((rule or rules) adj2 care).tw.	120
71	(standard? adj2 practi#e?).mp.	8841
72	surgical pathway?.mp.	130
73	(treat* adj2 protocol?).mp.	18585
74	(treatment adj2 plan*).mp.	55348
75	(treatment* adj2 (path or paths or pathway or pathways)).mp.	2933
76	or/1-75 [Care/Clinical/Critical Pathways & related terms]	731974
77	exp aged/ or "aged, 80 and over"/ or frail elderly/	3045838
78	exp Geriatrics/	29657
79	exp Geriatric Assessment/	26610
80	Geriatric Psychiatry/	2334
81	exp Health Services for the Aged/	17459
82	exp Geriatric Nursing/	13517
83	"older than 1##".mp.	95
84	"older than 6#".mp.	7047
85	"older than 7#".mp.	3197
86	"older than 8#".mp.	1197
87	"older than 9#".mp.	116
88	("over 1##" adj8 year?).mp.	2280
89	("over 6#" adj8 year?).mp.	10165
90	("over 7#" adj8 year?).mp.	4897
91	("over 8#" adj8 year?).mp.	2538
92	("over 9#" adj8 year?).mp.	885
93	((old?? or advance?) adj (age or aging or ageing)).mp.	72868
94	((old?? or elder?? or senior?) adj (patient? or citizen?? or person? or people or geriatric* or population?)).mp.	182134
95	(aged adj2 "10# years").mp.	936
96	(aged adj2 "6# years").mp.	37227
97	(aged adj2 "65 years").mp.	16440
98	(aged adj2 "7# years").mp.	22070
99	(aged adj2 "8# years").mp.	11889
100	(aged adj2 "9# years").mp.	3953

101	(elder* adj1 patient?).mp.	59120
102	(old adj age).mp.	25257
103	(old* adj1 patient?).mp.	70042
104	(older adult* or older client* or older patient* or older person* or older people).mp.	116733
105	centenarian*.mp.	1683
106	elder?.mp.	15160
107	elderly.mp.	220463
108	geriatri*.mp.	94528
109	grandfather*.mp.	1256
110	grandma??.mp.	121
111	grandmother*.mp.	2353
112	grandpa??.mp.	39
113	grandparent*.mp.	2783
114	nonagenarian*.mp.	1154
115	octagenarian*.mp.	37
116	oncogeriatri*.mp.	129
117	onco-geriatri*.mp.	23
118	orthogeriatri*.mp.	280
119	ortho-geriatri*.mp.	24
120	psychogeriatri*.mp.	1632
121	psycho-geriatri*.mp.	118
122	retiree*.mp.	1372
123	retirement?.mp.	16587
124	senior citizen*.mp.	1312
125	septuagenarian*.mp.	311
126	sexagenarian*.mp.	73
127	supercentenarian*.mp.	85
128	super-centenarian*.mp.	6
129	or/77-128 [Aged or Elderly & related terms]	3207290
130	76 and 129 [Care Pathways + Elderly]	112401
131	exp "Anesthesia and Analgesia"/	230059
132	exp Anesthesia Recovery Period/	5118
133	exp Anesthesia/	189037
134	exp anesthesiologists/	812
135	exp Anesthesiology/	30905
136	exp Anesthetics/	241114
137	exp Intraoperative care/	16647
138	exp Intraoperative Period/	26959
139	exp Perioperative Care/	147648
140	exp Perioperative Nursing/	13554
141	exp Perioperative Period/	86731

142	exp Postoperative care/	58785
143	exp Postoperative Complications/	534212
144	exp Preoperative care/	68496
145	exp Specialties, Surgical/	195865
146	exp Surgeons/	7591
147	exp Surgical Procedures, Operative/	3070334
148	Operating Rooms/	13477
149	Operative Time/	13088
150	Perioperative Nursing/	6651
151	Preanesthetic Medication/	7943
152	Preoperative Period/	6873
153	su.fs. [Surgery floating subheading]	1950426
154	(after adj6 (surgery or surgeries or surgical*)).mp.	283290
155	(before adj2 operat????).mp.	15291
156	(before adj3 procedur*).mp.	9083
157	(before adj6 (surgery or surgeries or surgical*)).mp.	67642
158	(during adj6 (surgery or surgeries or surgical*)).mp.	92440
159	(follow* adj6 (surgery or surgeries or surgical*)).mp.	108367
160	(operating adj2 room?).mp.	36306
161	(operating adj2 suite?).mp.	598
162	(operating adj2 theater?).mp.	972
163	(operating adj2 theatre?).mp.	3435
164	(operating adj2 unit?).mp.	547
165	(prior adj3 operat????).mp.	3401
166	(prior adj3 surgery).mp.	16934
167	(prior adj3 procedur*).mp.	3635
168	(undergo* adj6 (surgery or surgeries or surgical*)).mp.	89449
169	an?esth*.mp.	410054
170	intraoperat*.mp.	159334
171	intra-operat*.mp.	12017
172	operation?.mp.	372742
173	operative*.mp.	299952
174	peri*procedur*.mp.	4473
175	perioperat*.mp.	90361
176	peri-operat*.mp.	5917
177	periprocedur*.mp.	4473
178	peri-procedur*.mp.	904
179	peroperat*.mp.	4491
180	pos*ostomy.mp.	143
181	pos*otomy.mp.	2499
182	post*ectomies.mp.	3

183	post*ectomy.mp.	12120
184	post*otomies.mp.	0
185	post*otomy.mp.	2499
186	post*surger*.mp.	4814
187	post-intervention*.mp.	10894
188	postoperat*.mp.	764729
189	post-operat*.mp.	59859
190	postproced*.mp.	5970
191	post-proced*.mp.	5002
192	postsurgical*.mp.	12126
193	post-surgical*.mp.	6595
194	preintervention*.mp.	3228
195	pre-intervention*.mp.	3469
196	preoperat*.mp.	282052
197	pre-operat*.mp.	27339
198	preprocedur*.mp.	3032
199	pre-procedur*.mp.	1809
200	reoperat*.mp.	102555
201	re-operat*.mp.	4868
202	re-resect*.mp.	587
203	resect*.mp.	299288
204	or/131-203 [Perioperative - Pre- Intra- Post Operative & related terms]	4592296
205	130 and 204 [Care Pathways + Elderly + Periop]	32600
206	Clinical Trial, Phase III.pt.	16202
207	Clinical Trial, Phase III/	16202
208	Clinical Trial.pt.	520601
209	Clinical Trials, Phase III as Topic/	9152
210	Comparative Study.pt.	1852308
211	Comparative Study/	1852308
212	Controlled Clinical Trial.pt.	93514
213	Controlled Clinical Trial/	93514
214	Controlled Clinical Trials as Topic/	5484
215	Cross-Sectional Studies/	316329
216	Double-Blind Method/	155899
217	Equivalence Trial.pt.	485
218	Equivalence Trial/	485
219	Equivalence Trials as Topic/	261
220	Evaluation Studies.pt.	247852
221	exp Case-Control Studies/	1050987
222	exp Cohort Studies/	1949207
223	exp Randomized Controlled Trial/	499645

224	exp Randomized Controlled Trials as Topic/	133043
225	Longitudinal Studies/	130600
226	Meta-Analysis as Topic/	17568
227	Meta-Analysis/	110069
228	Multicenter Studies as Topic/	18103
229	Multicenter Study.pt.	265296
230	Multicenter Study/	265296
231	Placebos/	34703
232	Practice Guideline.pt.	25758
233	Pragmatic Clinical Trial.pt.	1284
234	Pragmatic Clinical Trial/	1284
235	Pragmatic Clinical Trials as Topic/	392
236	Prospective Studies/	527110
237	Randomized Controlled Trial.pt.	498915
238	Retrospective Studies/	795797
239	Systematic Review/ [New MeSH2019]	119887
240	Systematic Review.pt. [New PT 2019]	119887
241	Systematic Reviews as Topic/ [New MeSH2019]	3012
242	Validation Studies/	97924
243	Validation Studies.pt.	97924
244	("phase 3" or "phase3" or "phase III").mp.	49908
245	((multicenter* or multicentre*) adj2 (trial? or study or studies)).mp.	297925
246	((noninferiority or non-inferiority) adj4 (trial? or study or studies)).mp.	3674
247	((single or double or triple or treble) adj3 (blind* or mask*)).mp.	217323
248	(case control* adj2 (study or studies)).mp.	299641
249	(comparative adj2 (trial? or study or studies)).mp.	1900334
250	(conceal* adj2 allocat*).mp.	2478
251	(controlled adj1 clinical adj2 (trial? or study or studies)).mp.	123616
252	(cross-sectional* adj2 (study or studies)).mp.	337087
253	(equivalen* adj4 (trial? or study or studies)).mp.	4351
254	(evaluation adj1 (study or studies)).mp.	374799
255	(longitudinal* adj2 (study or studies)).mp.	162323
256	(meta-anal* or metanal* or metaanal*).mp.	161440
257	(overview? adj4 (review or reviews)).mp.	13715
258	(pragmatic adj2 (trial? or study or studies)).mp.	3208
259	(prospective* adj2 (study or studies)).mp.	607557
260	(retrospective* adj2 (study or studies)).mp.	825682
261	(superiority adj4 (trial? or study or studies)).mp.	2713
262	(systematic adj4 (review or reviews or overview or overviews)).mp.	146342
263	(validation adj1 (study or studies)).mp.	108677
264	cohort*.mp.	565834

265	placebo*.mp.	205300
266	quasirandom*.mp.	103
267	random*.mp.	1165504
268	or/206-267 [Studies]	5594666
269	exp Qualitative Research/	51390
270	Evaluation Studies/	247852
271	Feasibility Studies/	65566
272	Interview/	28164
273	Patient Health Questionnaire/	277
274	Grounded Theory/	1473
275	Hermeneutics/	271
276	Nursing Methodology Research/	16346
277	Observational Study/	73603
278	Observational Studies as Topic/	4639
279	Patient Satisfaction/	79792
280	Pilot Projects/	118901
281	Program Evaluation/	61516
282	"Surveys and Questionnaires"/	448137
283	((discourse* or discurs*) adj3 analys#s).tw.	1812
284	((purpos* adj4 sampl*) or (focus adj group*)).af.	50875
285	(account or accounts or unstructured or open-ended or open ended or text* or narrative*).mp.	500496
286	(action research or cooperative inquir* or co operative inquir* or co- operative inquir*).mp.	3493
287	(constant adj (comparative or comparison)).af.	3727
288	(corbin* adj2 strauss*).tw.	238
289	(emic or etic or hermeneutic* or heuristic* or semiotic*).af. or (data adj1 saturat*).tw. or participant observ*.tw.	18048
290	(evaluat* adj2 (study or studies)).mp.	519869
291	(field adj (study or studies or research)).tw.	13560
292	(grounded adj (theor* or study or studies or research or analys#s)).af.	9669
293	(humanistic or existential or experiential or paradigm*).mp.	124720
294	(life stor* or women* stor*).mp.	1118
295	(life world or life-world or conversation analys#s or personal experience* or theoretical saturation).mp.	13451
296	((lived or life) adj experience*).mp.	8694
297	(merleau adj ponty*).tw.	175
298	(observational adj (study or studies or research)).tw.	82106
299	(social construct* or (postmodern* or post- structural*) or (post structural* or poststructural*) or post modern* or post-modern* or feminis* or interpret*).mp.	450663
300	(survey? or surveyed or surveying).mp.	889485
301	(theme* or thematic).mp.	81482
302	(van adj kaam*).tw.	34
303	(van adj manen*).tw.	349
304	biographical method?.tw.	25

305	cluster sampl*.mp.	5911
306	colaizzi*.tw.	538
307	content analys#s.af.	21645
308	ethnograph*.mp.	8722
309	ethnological research.mp.	7
310	ethnonursing.af.	108
311	foucault*.tw.	659
312	glaser*.tw.	748
313	heidegger*.tw.	594
314	human science.tw.	227
315	narrative analys#s.af.	998
316	observational method*.af.	590
317	phenomenol*.af.	19114
318	qualitative.af.	193982
319	questionnaire*.mp.	641975
320	spiegelberg*.tw.	70
321	theoretical sampl*.af.	550
322	(client* adj2 (satisfaction or satisfied or satisfy*)).mp.	1160
323	(content analys* or thematic analys* or narrative analys*).mp.	35519
324	(ethnol* or ethnog* or ethnonurs* or emic or etic).mp.	168190
325	(feasib* adj2 (study or studies)).mp.	71361
326	(Grounded adj5 theor*).mp.	10644
327	(hermeneutic* or phenomenolog* or lived experience*).mp.	22961
328	(integrat* adj1 model?).mp.	4940
329	(meta-ethnog* or metaethnog* or meta-narrat* or metanarrat* or meta-interpret* or metainterpret*).mp.	582
330	(metasynthes* or meta-synthes* or metasummar* or meta-summar* or metastud* or meta-stud*).ti,ab.	1018
331	(multiple adj1 perspective?).mp.	774
332	(patient?? adj2 (satisfaction or satisfied or satisfy*)).mp.	98534
333	(personal adj1 (story or stories)).mp.	432
334	(personal adj1 account???).mp.	880
335	(program* adj3 evaluat*).mp.	77236
336	(qualitative adj5 metaanaly*).mp.	3
337	(qualitative adj5 meta-analy*).mp.	413
338	(therapeutic adj1 model?).mp.	763
339	(treatment? adj1 model?).mp.	3115
340	action research.ti,ab.	3209
341	contextual*.mp.	30180
342	focus group?.mp.	41102
343	frame work*.mp.	380
344	framework*.mp.	187177
345	giorgi*.mp.	573

346	interview?.mp.	279111
347	multimethod inquir*.mp.	1
348	multi-method inquir*.mp.	1
349	multiperspective?.mp.	101
350	multi-perspective?.mp.	124
351	narrative?.mp.	33191
352	phenomenological*.mp.	13132
353	qualitative*.mp.	227614
354	qualitative.mp.	192865
355	questionnaire?.mp.	641921
356	thematic.mp.	22984
357	theme.mp.	17278
358	themes.mp.	51929
359	or/269-358 [Qualitative Research & Related Terms]	3350898
360	268 or 359 [Quantitative OR Qualitative Studies]	7394838
361	205 and 360 [Care Pathways + Elderly + Periop + Studies]	25396
362	limit 361 to english language	23719
363	exp animals/ not (exp animals/ and exp humans/)	4666483
364	362 not 363	23716
365	limit 362 to humans	23709
366	364 or 365	23716
367	limit 366 to yr="2010 -Current"	15596
368	limit 367 to ("all infant (birth to 23 months)" or "all child (0 to 18 years)" or "newborn infant (birth to 1 month)" or "infant (1 to 23 months)" or "preschool child (2 to 5 years)" or "child (6 to 12 years)" or "adolescent (13 to 18 years)" or "young adult (19 to 24 years)" or "adult (19 to 44 years)" or "young adult and adult (19-24 and 19-44)" or "middle age (45 to 64 years)")	13410
369	367 not 368	2186
370	limit 367 to ("all aged (65 and over)" or "aged (80 and over)")	15058
371	369 or 370	15313
372	from 371 keep 1-5999	5999
373	remove duplicates from 372	5947
374	from 371 keep 6000-9999	4000
375	remove duplicates from 374	4000
376	from 371 keep 10000-15313	5314
377	remove duplicates from 376	5314
378	373 or 375 or 377	15261

Medline In-Process

Ovid MEDLINE(R) Epub Ahead of Print and In-Process & Other Non-Indexed Citations January 22, 2020

#	Searches	Results

1	Algorithms/ and ("Patient Care"/ or "Progressive Patient Care"/ or "Patient Care Planning"/ or "Patient Care Management"/ or "Continuity of Patient Care"/ or "Episode of Care"/)	0
2	Clinical Protocols/	0
3	Critical Pathways/	0
4	Decision Support Techniques/	0
5	Decision Theory/	0
6	exp Benchmarking/	0
7	exp Decision Trees/	0
8	exp Guideline/	568
9	exp Guidelines As Topic/	0
10	exp Practice Guideline/	545
11	exp Practice Guidelines As Topic/	0
12	Guideline Adherence/	0
13	Health planning guidelines/	0
14	Models, Organizational/	0
15	Patient Care Bundles/	0
16	Physician's Practice Patterns/	0
17	algorhythm*.mp.	4
18	algorism*.mp.	15
19	(algorithm* and care).mp.	2880
20	bench mark*.mp.	65
21	benchmark*.mp.	12231
22	decision tree?.mp.	1647
23	flow chart?.mp.	178
24	flow diagram???.mp.	146
25	flowchart?.mp.	230
26	((comply or complies or compliant or compliance) adj2 (policy or policies)).mp.	44
27	((comply or complies or compliant or compliance) adj2 protocol*).mp.	133
28	(best adj2 practi#e?).mp.	5558
29	(care adj (bundle or bundles)).mp.	198
30	"care intervention?".mp.	951
31	(care adj2 (path or paths or pathway or pathways)).mp.	1165
32	(care adj2 map*).mp.	50
33	care model?.mp.	1280
34	(care adj2 plan*).mp.	2693
35	(clinical adj1 (path or paths or pathway or pathways)).mp.	580
36	(clinical adj2 protocol?).mp.	1081
37	(comprehensive adj2 care).mp.	1064
38	(coordinated adj2 care).mp.	368
39	(co-ordinated adj2 care).mp.	23
40	(critical adj2 (path or paths or pathway or pathways)).mp.	738

41	(decision adj2 tree?).mp.	1668
42	(decision? adj2 aid?).mp.	941
43	(decision? adj2 analy*).mp.	1641
44	(decision? adj2 model*).mp.	1485
45	(decision? adj2 techni*).mp.	249
46	((framework? or frame work?) and care).mp.	6699
47	((guideline or guidelines) adj2 care).mp.	634
48	(guidance adj2 (introduc* or issu* or impact* or effect* or disseminat* or distribut* or implement*)).mp.	355
49	(management adj2 protocol*).mp.	877
50	(multicomponent adj2 (path? or pathway?)).mp.	10
51	(multi-component adj2 (path? or pathway?)).mp.	7
52	(multicomponent adj2 (program? or programme?)).mp.	68
53	(multi-component adj2 (program? or programme?)).mp.	20
54	(multimodal adj2 (path? or pathway?)).mp.	21
55	(multimodal adj2 (program? or programme?)).mp.	106
56	(multi-modal adj2 (program? or programme?)).mp.	5
57	(nurs* adj2 protocol*).mp.	107
58	(optimal* adj2 care).mp.	968
59	(optimi* adj2 care).mp.	806
60	optimi#ation.mp.	36284
61	(organi#ational adj1 model?).mp.	121
62	((policy or policies) adj2 care).tw.	703
63	(practi#e adj1 parameter?).mp.	69
64	(practi#e adj1 pattern?).tw.	1212
65	(practi#e adj2 (protocol* or policy or policies or guideline*)).mp.	6416
66	(proactive* adj2 care).mp.	57
67	(process?? adj2 (chart? or diagram* or flowchart*)).mp.	139
68	((program or programme) adj project?).mp.	45
69	((rule or rules) adj2 care).tw.	19
70	(standard? adj2 practi#e?).mp.	1550
71	surgical pathway?.mp.	38
72	(treat* adj2 protocol?).mp.	3212
73	(treatment adj2 plan*).mp.	9023
74	(treatment* adj2 (path or paths or pathway or pathways)).mp.	730
75	or/1-74 [Care/Clinical/Critical Pathways & related terms]	99221
76	exp aged/ or "aged, 80 and over"/ or frail elderly/	0
77	exp Geriatrics/	0
78	exp Geriatric Assessment/	0
79	Geriatric Psychiatry/	0
80	exp Health Services for the Aged/	0
81	exp Geriatric Nursing/	0

82	"older than 1##".mp.	9
83	"older than 6#".mp.	872
84	"older than 7#".mp.	315
85	"older than 8#".mp.	148
86	"older than 9#".mp.	21
87	("over 1##" adj8 year?).mp.	400
88	("over 6#" adj8 year?).mp.	1385
89	("over 7#" adj8 year?).mp.	523
90	("over 8#" adj8 year?).mp.	301
91	("over 9#" adj8 year?).mp.	137
92	((old?? or advance?) adj (age or aging or ageing)).mp.	10525
93	((old?? or elder?? or senior?) adj (patient? or citizen?? or person? or people or geriatric* or population?)).mp.	27819
94	(aged adj2 "10# years").mp.	143
95	(aged adj2 "6# years").mp.	6114
96	(aged adj2 "65 years").mp.	2956
97	(aged adj2 "7# years").mp.	2933
98	(aged adj2 "8# years").mp.	1697
99	(aged adj2 "9# years").mp.	504
100	(elder* adj1 patient?).mp.	8340
101	(old adj age).mp.	2975
102	(old* adj1 patient?).mp.	10494
103	(older adult* or older client* or older patient* or older person* or older people).mp.	23737
104	centenarian*.mp.	290
105	elder?.mp.	1954
106	elderly.mp.	28445
107	geriatri*.mp.	6231
108	grandfather*.mp.	159
109	grandma??.mp.	30
110	grandmother*.mp.	333
111	grandpa??.mp.	10
112	grandparent*.mp.	466
113	nonagenarian*.mp.	194
114	octagenarian*.mp.	4
115	oncogeriatri*.mp.	20
116	onco-geriatri*.mp.	9
117	orthogeriatri*.mp.	98
118	ortho-geriatri*.mp.	6
119	psychogeriatri*.mp.	128
120	psycho-geriatri*.mp.	8
121	retiree*.mp.	145
122	retirement?.mp.	2048

123	senior citizen*.mp.	146
124	septuagenarian*.mp.	54
125	sexagenarian*.mp.	20
126	supercentenarian*.mp.	11
127	super-centenarian*.mp.	0
128	or/76-127 [Aged or Elderly & related terms]	76401
129	75 and 128 [Care Pathways + Elderly]	3417
130	exp "Anesthesia and Analgesia"/	0
131	exp Anesthesia Recovery Period/	0
132	exp Anesthesia/	0
133	exp anesthesiologists/	0
134	exp Anesthesiology/	0
135	exp Anesthetics/	0
136	exp Intraoperative care/	0
137	exp Intraoperative Period/	0
138	exp Perioperative Care/	0
139	exp Perioperative Nursing/	0
140	exp Perioperative Period/	0
141	exp Postoperative care/	0
142	exp Postoperative Complications/	0
143	exp Preoperative care/	0
144	exp Specialties, Surgical/	2
145	exp Surgeons/	0
146	exp Surgical Procedures, Operative/	0
147	Operating Rooms/	0
148	Operative Time/	0
149	Perioperative Nursing/	0
150	Preanesthetic Medication/	0
151	Preoperative Period/	0
152	su.fs. [Surgery floating subheading]	0
153	(after adj6 (surgery or surgeries or surgical*)).mp.	41196
154	(before adj2 operat????).mp.	1214
155	(before adj3 procedur*).mp.	1312
156	(before adj6 (surgery or surgeries or surgical*)).mp.	9007
157	(during adj6 (surgery or surgeries or surgical*)).mp.	13435
158	(follow* adj6 (surgery or surgeries or surgical*)).mp.	17730
159	(operating adj2 room?).mp.	3892
160	(operating adj2 suite?).mp.	57
161	(operating adj2 theater?).mp.	159
162	(operating adj2 theatre?).mp.	435
163	(operating adj2 unit?).mp.	76

164	(prior adj3 operat????).mp.	343
165	(prior adj3 surgery).mp.	2990
166	(prior adj3 procedur*).mp.	614
167	(undergo* adj6 (surgery or surgeries or surgical*)).mp.	14161
168	an?esth*.mp.	35019
169	intraoperat*.mp.	17850
170	intra-operat*.mp.	2706
171	operation?.mp.	59503
172	operative*.mp.	36016
173	peri*procedur*.mp.	881
174	perioperat*.mp.	13229
175	peri-operat*.mp.	1078
176	periprocedur*.mp.	880
177	peri-procedur*.mp.	267
178	peroperat*.mp.	237
179	pos*ostomy.mp.	9
180	pos*otomy.mp.	166
181	post*ectomies.mp.	0
182	post*ectomy.mp.	992
183	post*otomies.mp.	1
184	post*otomy.mp.	166
185	post*surger*.mp.	711
186	post-intervention*.mp.	3431
187	postoperat*.mp.	62382
188	post-operat*.mp.	12664
189	postproced*.mp.	900
190	post-proced*.mp.	1384
191	postsurgical*.mp.	1909
192	post-surgical*.mp.	1493
193	preintervention*.mp.	648
194	pre-intervention*.mp.	991
195	preoperat*.mp.	33974
196	pre-operat*.mp.	5174
197	preprocedur*.mp.	531
198	pre-procedur*.mp.	512
199	reoperat*.mp.	3837
200	re-operat*.mp.	715
201	re-resect*.mp.	93
202	resect*.mp.	44606
203	or/130-202 [Perioperative - Pre- Intra- Post Operative & related terms]	253315
204	129 and 203 [Care Pathways + Elderly + Periop]	456

205	Clinical Trial, Phase III.pt.	1
206	Clinical Trial, Phase III/	1
207	Clinical Trial.pt.	433
208	Clinical Trials, Phase III as Topic/	0
209	Comparative Study.pt.	45
210	Comparative Study/	45
211	Controlled Clinical Trial.pt.	21
212	Controlled Clinical Trial/	21
213	Controlled Clinical Trials as Topic/	0
214	Cross-Sectional Studies/	0
215	Double-Blind Method/	0
216	Equivalence Trial.pt.	0
217	Equivalence Trial/	0
218	Equivalence Trials as Topic/	0
219	Evaluation Studies.pt.	26
220	exp Case-Control Studies/	1
221	exp Cohort Studies/	1
222	exp Randomized Controlled Trial/	277
223	exp Randomized Controlled Trials as Topic/	0
224	Longitudinal Studies/	0
225	Meta-Analysis as Topic/	0
226	Meta-Analysis/	69
227	Multicenter Studies as Topic/	0
228	Multicenter Study.pt.	4
229	Multicenter Study/	4
230	Placebos/	0
231	Practice Guideline.pt.	545
232	Pragmatic Clinical Trial.pt.	0
233	Pragmatic Clinical Trial/	0
234	Pragmatic Clinical Trials as Topic/	0
235	Prospective Studies/	0
236	Randomized Controlled Trial.pt.	277
237	Retrospective Studies/	1
238	Systematic Review/ [New MeSH2019]	631
239	Systematic Review.pt. [New PT 2019]	631
240	Systematic Reviews as Topic/ [New MeSH2019]	0
241	Validation Studies/	0
242	Validation Studies.pt.	0
243	("phase 3" or "phase3" or "phase III").mp.	6773
244	((multicenter* or multicentre*) adj2 (trial? or study or studies)).mp.	8676
245	((noninferiority or non-inferiority) adj4 (trial? or study or studies)).mp.	844

246	((single or double or triple or treble) adj3 (blind* or mask*)).mp.	16329
247	(case control* adj2 (study or studies)).mp.	13785
248	(comparative adj2 (trial? or study or studies)).mp.	15830
249	(conceal* adj2 allocat*).mp.	306
250	(controlled adj1 clinical adj2 (trial? or study or studies)).mp.	4015
251	(cross-sectional* adj2 (study or studies)).mp.	39309
252	(equivalen* adj4 (trial? or study or studies)).mp.	585
253	(evaluation adj1 (study or studies)).mp.	1000
254	(longitudinal* adj2 (study or studies)).mp.	13202
255	(meta-anal* or metanal* or metaanal*).mp.	33022
256	(overview? adj4 (review or reviews)).mp.	4155
257	(pragmatic adj2 (trial? or study or studies)).mp.	570
258	(prospective* adj2 (study or studies)).mp.	36971
259	(retrospective* adj2 (study or studies)).mp.	41015
260	(superiority adj4 (trial? or study or studies)).mp.	561
261	(systematic adj4 (review or reviews or overview or overviews)).mp.	38947
262	(validation adj1 (study or studies)).mp.	2840
263	cohort*.mp.	90722
264	placebo*.mp.	20108
265	quasirandom*.mp.	62
266	random*.mp.	167497
267	or/205-266 [Studies]	411358
268	exp Qualitative Research/	0
269	Evaluation Studies/	26
270	Feasibility Studies/	0
271	Interview/	732
272	Patient Health Questionnaire/	0
273	Grounded Theory/	0
274	Hermeneutics/	0
275	Nursing Methodology Research/	0
276	Observational Study/	93
277	Observational Studies as Topic/	0
278	Patient Satisfaction/	0
279	Pilot Projects/	0
280	Program Evaluation/	0
281	"Surveys and Questionnaires"/	0
282	((discourse* or discurs*) adj3 analys#s).tw.	320
283	((purpos* adj4 sampl*) or (focus adj group*)).af.	11204
284	(account or accounts or unstructured or open-ended or open ended or text* or narrative*).mp.	121818
285	(action research or cooperative inquir* or co operative inquir* or co- operative inquir*).mp.	634
286	(constant adj (comparative or comparison)).af.	700

287	(corbin* adj2 strauss*).tw.	84
288	(emic or etic or hermeneutic* or heuristic* or semiotic*).af. or (data adj1 saturat*).tw. or participant observ*.tw.	4596
289	(evaluat* adj2 (study or studies)).mp.	28156
290	(field adj (study or studies or research)).tw.	2411
291	(grounded adj (theor* or study or studies or research or analys#s)).af.	1784
292	(humanistic or existential or experiential or paradigm*).mp.	24592
293	(life stor* or women* stor*).mp.	199
294	(life world or life-world or conversation analys#s or personal experience* or theoretical saturation).mp.	1474
295	((lived or life) adj experience*).mp.	2207
296	(merleau adj ponty*).tw.	40
297	(observational adj (study or studies or research)).tw.	19252
298	(social construct* or (postmodern* or post- structural*) or (post structural* or poststructural*) or post modern* or post-modern* or feminis* or interpret*).mp.	64201
299	(survey? or surveyed or surveying).mp.	89400
300	(theme* or thematic).mp.	21487
301	(van adj kaam*).tw.	3
302	(van adj manen*).tw.	59
303	biographical method?.tw.	0
304	cluster sampl*.mp.	1243
305	colaizzi*.tw.	128
306	content analys#s.af.	5731
307	ethnograph*.mp.	1835
308	ethnological research.mp.	0
309	ethnonursing.af.	5
310	foucault*.tw.	129
311	glaser*.tw.	228
312	heidegger*.tw.	52
313	human science.tw.	15
314	narrative analys#s.af.	208
315	observational method*.af.	151
316	phenomenol*.af.	6651
317	qualitative.af.	42297
318	questionnaire*.mp.	73327
319	spiegelberg*.tw.	9
320	theoretical sampl*.af.	123
321	(client* adj2 (satisfaction or satisfied or satisfy*)).mp.	175
322	(content analys* or thematic analys* or narrative analys*).mp.	10775
323	(ethnol* or ethnog* or ethnonurs* or emic or etic).mp.	2113
324	(feasib* adj2 (study or studies)).mp.	3925
325	(Grounded adj5 theor*).mp.	2118
326	(hermeneutic* or phenomenolog* or lived experience*).mp.	7646

327	(integrat* adj1 model?).mp.	1055
328	(meta-ethnog* or metaethnog* or meta-narrat* or metanarrat* or meta-interpret* or metainterpret*).mp.	150
329	(metasynthes* or meta-synthes* or metasummar* or meta-summar* or metastud* or meta-stud*).ti,ab.	310
330	(multiple adj1 perspective?).mp.	190
331	(patient?? adj2 (satisfaction or satisfied or satisfy*)).mp.	7908
332	(personal adj1 (story or stories)).mp.	81
333	(personal adj1 account???).mp.	247
334	(program* adj3 evaluat*).mp.	3083
335	(qualitative adj5 metaanaly*).mp.	0
336	(qualitative adj5 meta-analy*).mp.	122
337	(therapeutic adj1 model?).mp.	109
338	(treatment? adj1 model?).mp.	533
339	action research.ti,ab.	584
340	contextual*.mp.	7222
341	focus group?.mp.	8335
342	frame work*.mp.	104
343	framework*.mp.	74818
344	giorgi*.mp.	108
345	interview?.mp.	39445
346	multimethod inquir*.mp.	0
347	multi-method inquir*.mp.	1
348	multiperspective?.mp.	29
349	multi-perspective?.mp.	37
350	narrative?.mp.	9230
351	phenomenological*.mp.	4872
352	qualitative*.mp.	50665
353	qualitative.mp.	41915
354	questionnaire?.mp.	73290
355	thematic.mp.	7416
356	theme.mp.	4318
357	themes.mp.	13389
358	or/268-357 [Qualitative Research & Related Terms]	516602
359	267 or 358 [Quantitative OR Qualitative Studies]	816167
360	204 and 359 [Care Pathways + Elderly + Periop + Studies]	223
361	limit 360 to english language	218
362	remove duplicates from 361	218

Embase

Embase 1974 to 2020 January 22

#	Searches	Results
1	Algorithms/ and (patient care/ or collaborative care team/ or patient care planning/)	2251
2	Clinical Protocols/	92646
3	Critical Pathways/	8323
4	Decision Support Techniques/	18365
5	Decision Theory/	1711
6	exp Benchmarking/	4502
7	exp Decision Trees/	12170
8	exp Guidelines As Topic/	533387
9	exp Practice Guideline/	533387
10	exp Practice Guidelines As Topic/	533387
11	Guideline Adherence/	9499
12	Health planning guidelines/	92421
13	Models, Organizational/	45162
14	Patient Care Bundles/	1218
15	Physician's Practice Patterns/	226531
16	"care intervention?".ti,ab.	7509
17	((comply or complies or compliant or compliance) adj2 (policy or policies)).ti,ab.	393
18	((comply or complies or compliant or compliance) adj2 protocol*).ti,ab.	1578
19	((framework? or frame work?) and care).ti,ab.	46113
20	((guideline or guidelines) adj2 care).ti,ab.	6514
21	((policy or policies) adj2 care).tw.	7077
22	((program or programme) adj project?).ti,ab.	978
23	((rule or rules) adj2 care).tw.	178
24	((workflow* or work-flow*) adj2 map*4).mp.	132
25	((workflow* or work-flow*) adj2 process???).mp.	1083
26	(best adj2 practi#e?).ti,ab.	40518
27	(care adj (bundle or bundles)).ti,ab.	1444
28	(care adj2 (path or paths or pathway or pathways)).ti,ab.	9545
29	(care adj2 map*).ti,ab.	592
30	(care adj2 plan*).ti,ab.	27564
31	(clinical adj1 (path or paths or pathway or pathways)).ti,ab.	6089
32	(clinical adj2 protocol?).ti,ab.	11858
33	(comprehensive adj2 care).ti,ab.	11169
34	(coordinated adj2 care).ti,ab.	2756
35	(co-ordinated adj2 care).ti,ab.	170
36	(critical adj2 (path or paths or pathway or pathways)).ti,ab.	7832
37	(decision adj2 tree?).ti,ab.	12238

38	(decision? adj2 aid?).ti,ab.	7492
39	(decision? adj2 analy*).ti,ab.	15539
40	(decision? adj2 model*).ti,ab.	13549
41	(decision? adj2 techni*).ti,ab.	1039
42	(guidance adj2 (introduc* or issu* or impact* or effect* or disseminat* or distribut* or implement*)).ti,ab.	2972
43	(management adj2 protocol*).ti,ab.	7140
44	(multicomponent adj2 (path? or pathway?)).ti,ab.	42
45	(multi-component adj2 (path? or pathway?)).ti,ab.	26
46	(multicomponent adj2 (program? or programme?)).ti,ab.	451
47	(multi-component adj2 (program? or programme?)).ti,ab.	208
48	(multimodal adj2 (path? or pathway?)).ti,ab.	138
49	(multimodal adj2 (program? or programme?)).ti,ab.	790
50	(multi-modal adj2 (program? or programme?)).ti,ab.	124
51	(nurs* adj2 protocol*).ti,ab.	1249
52	(optimal* adj2 care).ti,ab.	9861
53	(optimi* adj2 care).ti,ab.	6745
54	(organi#ational adj1 model?).ti,ab.	1150
55	(practi#e adj1 parameter?).ti,ab.	1585
56	(practi#e adj1 pattern?).tw.	12497
57	(practi#e adj2 (protocol* or policy or policies or guideline*)).ti,ab.	47929
58	(proactive* adj2 care).ti,ab.	593
59	(process?? adj2 (chart? or diagram* or flowchart* or map*4)).ti,ab.	3804
60	((rule or rules) and care).ti,ab.	16683
61	(standard? adj2 practi#e?).ti,ab.	16361
62	(treat* adj2 protocol?).ti,ab.	33395
63	(treatment adj2 plan*).ti,ab.	99630
64	(treatment* adj2 (path or paths or pathway or pathways)).ti,ab.	6393
65	algorhythm*.ti,ab.	268
66	algorism*.ti,ab.	232
67	(algorithm* and care).ti,ab.	24438
68	bench mark*.ti,ab.	602
69	benchmark*.ti,ab.	46834
70	care model?.ti,ab.	9851
71	decision tree?.ti,ab.	12027
72	flow chart?.ti,ab.	2375
73	flow diagram???.ti,ab.	978
74	flowchart?.ti,ab.	1901
75	gold standard?.ti,ab.	106096
76	optimi#ation.ti,ab.	171149
77	surgical pathway?.ti,ab.	277
78	algorithm/ and (patient care/ or collaborative care team/ or patient care planning/) [Embase]	3132

79	care bundle/ [Embase]	1218
80	clinical decision support system/ [Embase]	2775
81	clinical pathway/ [Embase]	8323
82	clinical protocol/ [Embase]	96807
83	decision support system/ [Embase]	21703
84	"decision tree"/ [Embase]	12170
85	good clinical practice/ [Embase]	9124
86	health care planning/ [Embase]	96285
87	nursing care plan/ [Embase]	196
88	nursing protocol/ [Embase]	162
89	practice guideline/ [Embase]	405697
90	protocol compliance/ [Used for Guideline Adherence in Embase]	12394
91	or/1-90 [Algorithms or Pathways]	1564328
92	"Aged, 80 and Over"/	111308
93	Frail Elderly/	9822
94	exp Geriatrics/	36178
95	exp Gerontology/	2968
96	Geriatric Psychiatry/	7418
97	exp Health Services for the Aged/	74687
98	exp Geriatric Nursing/	12128
99	"older than 1##".ti,ab.	143
100	"older than 6#".ti,ab.	12380
101	"older than 7#".ti,ab.	5687
102	"older than 8#".ti,ab.	2315
103	"older than 9#".ti,ab.	230
104	("over 1##" adj8 year?).ti,ab.	3705
105	("over 6#" adj8 year?).ti,ab.	17749
106	("over 7#" adj8 year?).ti,ab.	8647
107	("over 8#" adj8 year?).ti,ab.	4834
108	("over 9#" adj8 year?).ti,ab.	1652
109	((old?? or advance?) adj (age or aging or ageing)).ti,ab.	115538
110	((old?? or elder?? or senior?) adj (patient? or citizen?? or person? or people or geriatric* or population?)).ti,ab.	296082
111	(aged adj2 "10# years").ti,ab.	1589
112	(aged adj2 "6# years").ti,ab.	59071
113	(aged adj2 "65 years").ti,ab.	27198
114	(aged adj2 "7# years").ti,ab.	35063
115	(aged adj2 "8# years").ti,ab.	19348
116	(aged adj2 "9# years").ti,ab.	6276
117	(elder* adj1 patient?).ti,ab.	102417
118	(old adj age).ti,ab.	32552
119	(old* adj1 patient?).ti,ab.	120702

120	(older adult* or older client* or older patient* or older person* or older people).ti,ab.	183115
121	centenarian*.ti,ab.	2288
122	elder?.ti,ab.	22611
123	elderly.ti,ab.	333334
124	geriatri*.ti,ab.	71566
125	gerontol*.ti,ab.	10854
126	grandfather*.ti,ab.	2236
127	grandma??.ti,ab.	181
128	grandmother*.ti,ab.	3914
129	grandpa??.ti,ab.	58
130	grandparent*.ti,ab.	3817
131	nonagenarian*.ti,ab.	1856
132	octagenarian*.ti,ab.	86
133	oncogeriatri*.ti,ab.	212
134	onco-geriatri*.ti,ab.	64
135	orthogeriatri*.ti,ab.	684
136	ortho-geriatri*.ti,ab.	69
137	psychogeriatri*.ti,ab.	3812
138	psycho-geriatri*.ti,ab.	164
139	retiree*.ti,ab.	1805
140	retirement?.ti,ab.	15275
141	senior citizen*.ti,ab.	1804
142	septuagenarian*.ti,ab.	514
143	sexagenarian*.ti,ab.	100
144	supercentenarian*.ti,ab.	100
145	super-centenarian*.ti,ab.	8
146	aged/ or aged hospital patient/ or frail elderly/ or institutionalized elderly/ or very elderly/ [Embase]	2897287
147	elderly care/ or exp geriatric care/ or home for the aged/ [Embase]	74309
148	geriatric care/ or geriatric hospital/ or geriatric nursing/ or geriatric patient/ or geriatric surgery/ or geriatrician/ or geriatrics/ [Embase]	78626
149	gerontologic nurse practitioner/ or gerontological research/ or gerontologist/ or gerontology/ [Embase]	3982
150	or/92-149 [Aged or Elderly or >=65 years of age]	3243640
151	91 and 150 [Care Pathway + Elderly]	172423
152	anesthesist/	24971
153	exp "Anesthesia and Analgesia"/	712277
154	exp Anesthesia Recovery Period/	7727
155	exp *Anesthesia/	155069
156	exp anesthesiologists/	5004
157	exp Anesthesiology/	18351
158	exp *anesthetic agent/	225176
159	exp anesthetic recovery/	7727

160	exp *Anesthetics/	225176
161	exp Intraoperative care/	12609
162	exp Intraoperative Period/	185435
163	exp Perioperative Care/	47812
164	exp Perioperative Nursing/	5821
165	exp Perioperative Period/	47812
166	exp peroperative care/	12609
167	exp peroperative complication/	43375
168	exp Postoperative care/	83960
169	exp Postoperative Complications/	647568
170	exp Preoperative care/	38002
171	exp Specialties, Surgical/	4643524
172	exp surgeon/	146933
173	exp Surgeons/	146933
174	exp *surgery/	2258154
175	exp *Surgical Procedures, Operative/	2258154
176	Operating Rooms/	33179
177	Operative Time/	72851
178	Perioperative Nursing/	5821
179	Preanesthetic Medication/	17222
180	Preoperative Period/	51764
181	su.fs.	1992965
182	(after adj6 (surgery or surgeries or surgical*)).ti,ab.	452175
183	(before adj2 operat????).ti,ab.	23665
184	(before adj2 surgery).ti,ab.	64418
185	(before adj3 procedur*).ti,ab.	17340
186	(before adj3 procedur*).ti,ab.	17340
187	(before adj6 (surgery or surgeries or surgical*)).ti,ab.	108661
188	(during adj6 (surgery or surgeries or surgical*)).ti,ab.	145267
189	(follow* adj6 (surgery or surgeries or surgical*)).ti,ab.	182039
190	(operating adj2 room?).ti,ab.	37080
191	(operating adj2 suite?).ti,ab.	857
192	(operating adj2 theater?).ti,ab.	1490
193	(operating adj2 theatre?).ti,ab.	5565
194	(operating adj2 unit?).ti,ab.	875
195	(prior adj3 operat????).ti,ab.	5700
196	(prior adj3 procedur*).ti,ab.	8814
197	(prior adj3 surgery).ti,ab.	36656
198	(undergo* adj6 (surgery or surgeries or surgical*)).ti,ab.	153333
199	an?esth*.ti,ab.	465480
200	intraoperat*.ti,ab.	182602

201	intra-operat*.ti,ab.	27933
202	operation?.ti,ab.	545804
203	operative*.ti,ab.	416078
204	peri*procedur*.ti,ab.	9597
205	perioperat*.ti,ab.	133744
206	peri-operat*.ti,ab.	14960
207	periprocedur*.ti,ab.	9594
208	peri-procedur*.ti,ab.	3596
209	peroperat*.ti,ab.	5261
210	pos*ostomy.ti,ab.	193
211	pos*otomy.ti,ab.	3101
212	post*ectomies.ti,ab.	5
213	post*ectomy.ti,ab.	12135
214	post*otomies.ti,ab.	1
215	post*otomy.ti,ab.	3100
216	post*surger*.ti,ab.	6909
217	post-intervention*.ti,ab.	24582
218	postoperat*.ti,ab.	685128
219	post-operat*.ti,ab.	137062
220	postproced*.ti,ab.	10092
221	post-proced*.ti,ab.	20202
222	postsurgical*.ti,ab.	17821
223	post-surgical*.ti,ab.	15362
224	preintervention*.ti,ab.	4743
225	pre-intervention*.ti,ab.	8551
226	preoperat*.ti,ab.	384435
227	pre-operat*.ti,ab.	61135
228	preprocedur*.ti,ab.	5656
229	pre-procedur*.ti,ab.	7582
230	reoperat*.ti,ab.	49168
231	re-operat*.ti,ab.	10051
232	re-resect*.ti,ab.	1191
233	reresect*.ti,ab.	289
234	resect*.ti,ab.	488019
235	or/152-234 [Periop Postop Preop Surgery or Anesthesia - Embase]	6237663
236	151 and 235 [Care Pathway + Elderly + Periop]	53522
237	case series.mp,kw.	99165
238	cohort analysis/	544276
239	cohort*.mp,kw.	1054911
240	Cross-Sectional Studies/	205035
241	cross-sectional study/	332577

242	double blind procedure/	169062
243	Double-Blind Method/	144667
244	doubleblind*.mp,kw.	3217
245	Longitudinal Studies/	115061
246	Placebo*.mp,kw.	447723
247	placebo/	346413
248	Placebos/	290014
249	Prospective Studies/	472105
250	exp Randomized controlled trial/	588839
251	exp Randomized Controlled Trials as Topic/	173460
252	"randomized controlled trial (topic)"/ [embase]	173460
253	random*.mp,kw.	1714197
254	ct.fs. [clinical trial]	619261
255	controlled clinical trial/	463373
256	Controlled Clinical Trials As Topic/	9247
257	"controlled clinical trial (topic)"/	10546
258	meta analysis/	179827
259	meta-analysis as topic/	28337
260	"meta-analysis (topic)"/	41180
261	Pragmatic Clinical Trial/	646
262	Pragmatic Clinical Trials As Topic/	173460
263	systematic review/	232105
264	"systematic review (topic)"/	24415
265	validation study/	81488
266	evaluation study/	43637
267	exp case control study/	169123
268	exp Case-Control Studies/	169123
269	exp Cohort Studies/	544276
270	((single or double or treble or triple) adj3 (blind* or mask*)).mp,kw.	302021
271	(case control* adj2 (study or studies)).mp,kw.	213537
272	(controlled adj1 clinical adj2 (trial? or study or studies)).mp,kw.	500167
273	(cross-sectional* adj2 (study or studies)).mp,kw.	394922
274	(evaluation adj1 (study or studies)).mp,kw.	52153
275	(longitudinal* adj2 (study or studies)).mp,kw.	184792
276	(meta-anal* or metanal* or metaanal*).mp,kw.	287925
277	(overview? adj4 (review or reviews)).mp,kw.	18943
278	(pragmatic adj2 (trial? or study or studies)).mp,kw.	3751
279	(prospective* adj2 (study or studies)).mp,kw.	758471
280	(systematic adj4 (review or reviews or overview or overviews)).mp,kw.	314632
281	(validation adj1 (study or studies)).mp,kw.	91868
282	(content analys* or thematic analys* or narrative analys*).mp.	59165

283	(ethnol* or ethnog* or ethnonurs* or emic or etic).mp.	84313
284	(grounded adj5 theor*).mp,kw.	15835
285	(hermeneutic* or phenomenolog* or lived experience*).mp,kw.	37978
286	(meta-ethnog* or metaethnog* or meta-narrat* or metanarrat* or meta-interpret* or metainterpret*).mp,kw.	826
287	(metasynthes* or meta-synthes* or metasummar* or meta-summar* or metastud* or meta-stud*).mp,kw.	1536
288	action research.mp,kw.	5190
289	exp qualitative research/	71315
290	giorgi*.mp,kw.	842
291	nursing methodology research/	14740
292	qualitative.mp,kw.	309114
293	quasirandom*.mp.	211
294	or/237-293	5021595
295	236 and 294 [Care Pathway + Elderly + Periop + Studies]	21998
296	limit 295 to english language	21327
297	(exp animals/ or exp animal experimentation/ or nonhuman/) not ((exp animals/ or exp animal experimentation/ or nonhuman/) and exp human/)	6380470
298	296 not 297	21299
299	limit 296 to human	21163
300	298 or 299	21299
301	limit 300 to (embryo <first trimester> or infant <to one year> or child <unspecified age> or preschool child <1 to 6 years> or school child <7 to 12 years> or adolescent <13 to 17 years> or adult <18 to 64 years>)	14392
302	300 not 301	6907
303	limit 300 to aged <65+ years>	18721
304	302 or 303	20509
305	limit 304 to (conference abstracts or conference abstract status or (abstract report or books or "book review" or chapter or conference abstract or "conference review") or (book or book series or conference proceeding))	2770
306	conferenc*.so.	546642
307	305 or 306	549113
308	304 not 307	17737
309	limit 308 to yr="2010 -Current"	11954
310	medline.cr.	7872691
311	309 not 310	10244
312	from 311 keep 1-5999	5999
313	remove duplicates from 312	5951
314	from 311 keep 6000-10244	4245
315	remove duplicates from 314	4218
316	313 or 315	10169

CCTR

Cochrane Central Register of Controlled Trials 2014 to Present

#	Searches	Results
1	Algorithms/ and ("Patient Care"/ or "Progressive Patient Care"/ or "Patient Care Planning"/ or "Patient Care Management"/ or "Continuity of Patient Care"/ or "Episode of Care"/)	25
2	Clinical Protocols/	4677
3	Critical Pathways/	189
4	Decision Support Techniques/	774
5	Decision Theory/	6
6	exp Benchmarking/	99
7	exp Decision Trees/	159
8	exp Guideline/	0
9	exp Guidelines As Topic/	1844
10	exp Practice Guideline/	0
11	exp Practice Guidelines As Topic/	1576
12	Guideline Adherence/	1026
13	Health planning guidelines/	12
14	Models, Organizational/	161
15	Patient Care Bundles/	23
16	Physician's Practice Patterns/	0
17	algorhythm*.ti,ab.	6
18	algorism*.ti,ab.	16
19	(algorithm* and care).ti,ab.	2444
20	bench mark*.ti,ab.	28
21	benchmark*.ti,ab.	1237
22	decision tree?.ti,ab.	509
23	flow chart?.ti,ab.	163
24	flow diagram???.ti,ab.	83
25	flowchart?.ti,ab.	115
26	((comply or complies or compliant or compliance) adj2 (policy or policies)).ti,ab.	33
27	((comply or complies or compliant or compliance) adj2 protocol*).ti,ab.	2299
28	(best adj2 practi#e?).ti,ab.	2096
29	(care adj (bundle or bundles)).ti,ab.	118
30	"care intervention?".ti,ab.	3923
31	(care adj2 (path or paths or pathway or pathways)).ti,ab.	775
32	(care adj2 map*).ti,ab.	80
33	care model?.ti,ab.	1332
34	(care adj2 plan*).ti,ab.	2243
35	(clinical adj1 (path or paths or pathway or pathways)).ti,ab.	487
36	(clinical adj2 protocol?).ti,ab.	1902
37	(comprehensive adj2 care).ti,ab.	773
38	(coordinated adj2 care).ti,ab.	255
39	(co-ordinated adj2 care).ti,ab.	15

40	(critical adj2 (path or paths or pathway or pathways)).ti,ab.	146
41	(decision adj2 tree?).ti,ab.	521
42	(decision? adj2 aid?).ti,ab.	1542
43	(decision? adj2 analy*).ti,ab.	1107
44	(decision? adj2 model*).ti,ab.	1022
45	(decision? adj2 techni*).ti,ab.	75
46	((framework? or frame work?) and care).ti,ab.	2087
47	((guideline or guidelines) adj2 care).ti,ab.	1023
48	(guidance adj2 (introduc* or issu* or impact* or effect* or disseminat* or distribut* or implement*)).ti,ab.	450
49	(management adj2 protocol*).ti,ab.	1002
50	(multicomponent adj2 (path? or pathway?)).ti,ab.	1
51	(multi-component adj2 (path? or pathway?)).ti,ab.	1
52	(multicomponent adj2 (program? or programme?)).ti,ab.	213
53	(multi-component adj2 (program? or programme?)).ti,ab.	120
54	(multimodal adj2 (path? or pathway?)).ti,ab.	20
55	(multimodal adj2 (program? or programme?)).ti,ab.	290
56	(multi-modal adj2 (program? or programme?)).ti,ab.	53
57	(nurs* adj2 protocol*).ti,ab.	255
58	(optimal* adj2 care).ti,ab.	569
59	(optimi* adj2 care).ti,ab.	634
60	optimi#ation.ti,ab.	5045
61	(organi#ational adj1 model?).ti,ab.	38
62	((policy or policies) adj2 care).tw.	349
63	(practi#e adj1 parameter?).ti,ab.	67
64	(practi#e adj1 pattern?).tw.	562
65	(practi#e adj2 (protocol* or policy or policies or guideline*)).ti,ab.	3330
66	(proactive* adj2 care).ti,ab.	123
67	(process?? adj2 (chart? or diagram* or flowchart*)).ti,ab.	47
68	((program or programme) adj project?).ti,ab.	114
69	((rule or rules) adj2 care).tw.	20
70	(standard? adj2 practi#e?).ti,ab.	2580
71	surgical pathway?.ti,ab.	12
72	(treat* adj2 protocol?).ti,ab.	8911
73	(treatment adj2 plan*).ti,ab.	5226
74	(treatment* adj2 (path or paths or pathway or pathways)).ti,ab.	603
75	or/1-74 [Care/Clinical/Critical Pathways & related terms]	57513
76	exp aged/ or "aged, 80 and over"/ or frail elderly/	202606
77	exp Geriatrics/	199
78	exp Geriatric Assessment/	1454
79	Geriatric Psychiatry/	39
80	exp Health Services for the Aged/	444

81	exp Geriatric Nursing/	176
82	"older than 1##".ti,ab.	92
83	"older than 6#".ti,ab.	1415
84	"older than 7#".ti,ab.	628
85	"older than 8#".ti,ab.	188
86	"older than 9#".ti,ab.	36
87	("over 1##" adj8 year?).ti,ab.	191
88	("over 6#" adj8 year?).ti,ab.	1626
89	("over 7#" adj8 year?).ti,ab.	663
90	("over 8#" adj8 year?).ti,ab.	244
91	("over 9#" adj8 year?).ti,ab.	98
92	((old?? or advance?) adj (age or aging or ageing)).ti,ab.	4883
93	((old?? or elder?? or senior?) adj (patient? or citizen?? or person? or people or geriatric* or population?)).ti,ab.	24499
94	(aged adj2 "10# years").ti,ab.	151
95	(aged adj2 "6# years").ti,ab.	14159
96	(aged adj2 "65 years").ti,ab.	7359
97	(aged adj2 "7# years").ti,ab.	10234
98	(aged adj2 "8# years").ti,ab.	5039
99	(aged adj2 "9# years").ti,ab.	823
100	(elder* adj1 patient?).ti,ab.	11146
101	(old adj age).ti,ab.	1084
102	(old* adj1 patient?).ti,ab.	8564
103	(older adult* or older client* or older patient* or older person* or older people).ti,ab.	18817
104	centenarian*.ti,ab.	24
105	elder?.ti,ab.	1714
106	elderly.ti,ab.	41705
107	geriatri*.ti,ab.	5740
108	grandfather*.ti,ab.	12
109	grandma??.ti,ab.	2
110	grandmother*.ti,ab.	88
111	grandpa??.ti,ab.	1
112	grandparent*.ti,ab.	111
113	nonagenarian*.ti,ab.	43
114	octagenarian*.ti,ab.	1
115	oncogeriatri*.ti,ab.	10
116	onco-geriatri*.ti,ab.	3
117	orthogeriatri*.ti,ab.	44
118	ortho-geriatri*.ti,ab.	6
119	psychogeriatri*.ti,ab.	231
120	psycho-geriatri*.ti,ab.	22
121	retiree*.ti,ab.	40

122	retirement?.ti,ab.	377
123	senior citizen*.ti,ab.	125
124	septuagenarian*.ti,ab.	19
125	sexagenarian*.ti,ab.	2
126	supercentenarian*.ti,ab.	0
127	super-centenarian*.ti,ab.	0
128	or/76-127 [Aged or Elderly & related terms]	272268
129	75 and 128 [Care Pathways + Elderly]	11843
130	exp "Anesthesia and Analgesia"/	26100
131	exp Anesthesia Recovery Period/	1999
132	exp Anesthesia/	18500
133	exp Anesthesiology/	408
134	exp Anesthetics/	31079
135	exp Intraoperative care/	1542
136	exp Intraoperative Period/	2431
137	exp Perioperative Care/	11865
138	exp Perioperative Nursing/	124
139	exp Perioperative Period/	8205
140	exp Postoperative care/	4371
141	exp Postoperative Complications/	37229
142	exp Preoperative care/	5762
143	exp Specialties, Surgical/	1788
144	exp Barber Surgeons/	0
145	exp Surgical Procedures, Operative/	112707
146	Operating Rooms/	209
147	Operative Time/	1208
148	Perioperative Nursing/	66
149	Preanesthetic Medication/	1714
150	Preoperative Period/	261
151	su.fs. [Surgery floating subheading]	56036
152	(after adj6 (surgery or surgeries or surgical*)).ti,ab.	185502
153	(before adj2 operat????).ti,ab.	84621
154	(before adj3 procedur*).ti,ab.	100715
155	(before adj6 (surgery or surgeries or surgical*)).ti,ab.	185502
156	(during adj6 (surgery or surgeries or surgical*)).ti,ab.	185502
157	(follow* adj6 (surgery or surgeries or surgical*)).ti,ab.	5625
158	(operating adj2 room?).ti,ab.	3868
159	(operating adj2 suite?).ti,ab.	64
160	(operating adj2 theater?).ti,ab.	153
161	(operating adj2 theatre?).ti,ab.	482
162	(operating adj2 unit?).ti,ab.	61

163	(prior adj3 operat????).ti,ab.	855
164	(prior adj3 surgery).ti,ab.	5333
165	(prior adj3 procedur*).ti,ab.	2776
166	(undergo* adj6 (surgery or surgeries or surgical*)).ti,ab.	31556
167	an?esth*.ti,ab.	70534
168	intraoperat*.ti,ab.	19900
169	intra-operat*.ti,ab.	3427
170	operation?.ti,ab.	35404
171	operative*.ti,ab.	38358
172	peri*procedur*.ti,ab.	1188
173	perioperat*.ti,ab.	16346
174	peri-operat*.ti,ab.	2007
175	periprocedur*.ti,ab.	1188
176	peri-procedur*.ti,ab.	410
177	peroperat*.ti,ab.	664
178	pos*ostomy.ti,ab.	8
179	pos*otomy.ti,ab.	317
180	post*ectomies.ti,ab.	0
181	post*ectomy.ti,ab.	955
182	post*otomies.ti,ab.	0
183	post*otomy.ti,ab.	317
184	post*surger*.ti,ab.	890
185	post-intervention*.ti,ab.	11757
186	postoperat*.ti,ab.	89126
187	post-operat*.ti,ab.	19934
188	postproced*.ti,ab.	1298
189	post-proced*.ti,ab.	2460
190	postsurgical*.ti,ab.	1813
191	post-surgical*.ti,ab.	1817
192	preintervention*.ti,ab.	1170
193	pre-intervention*.ti,ab.	2614
194	preoperat*.ti,ab.	33251
195	pre-operat*.ti,ab.	6091
196	preprocedur*.ti,ab.	582
197	pre-procedur*.ti,ab.	786
198	reoperat*.ti,ab.	2687
199	re-operat*.ti,ab.	760
200	re-resect*.ti,ab.	37
201	resect*.ti,ab.	25082
202	or/130-201 [Perioperative]	391070
203	129 and 202 [Care Pathways + Elderly + Perioperative]	3646

204	(animal or animals or ape or apes or baboon or baboons or bonobo or bonobos or cat or cats or chimpanzee or chimpanzees or dog or dogs or feline or felines or ferret or ferrets or goat or goats or horse or horses or lamb or lambs or macaque or macaques or mandrill or mandrills or mice or mink or minks or monkeys or monkeys or mouse or murine or pig or pigs or porcine or orangutan or orangutans or rat or rats or rodent or rodents or sheep or tamarin or tamarins or veterinary or veterinarian or veterinarians or weasel or weasels or veterinar*).ti.	3887
205	203 not 204	3644
206	(adolescence or adolescent or adolescents or babies or baby or boy or boys or child or childhood or children or fetus or fetal or foetus or foetal or girl or girls or infancy or infant or infants or neonatal or neonatally or neonate or neonates or newborn or newborns or paediatric or paediatrician or paediatricians or paediatrics or pediatric or pediatrician or pediatricians or pediatrics or teen or teenage or teenagers or teens or toddler or toddlers or youth or youths).ti.	122591
207	205 not 206	3604
208	(book or book article or book book or book note or "book review" or book series article or book series article in press or book series chapter or book series conference paper or book series letter or "book series review" or book series short survey or chapter or conference abstract or conference abstract placebo controlled partly blinded crossover study in 12 sle patients or conference proceeding or "conference review" or journal conference abstract or "journal conference review").pt.	166083
209	conferenc*.so.	42558
210	208 or 209	167987
211	207 not 210	3471
212	limit 211 to english language	2694
213	limit 212 to yr="2010 -Current"	1743
214	remove duplicates from 213	1711

CDSR

Cochrane Database of Systematic Reviews 2005 to Present

#	Searches	Results
1	algorhythm*.ti,ab.	0
2	algorism*.ti,ab.	0
3	algorithm*.ti,ab.	26
4	bench mark*.ti,ab.	0
5	benchmark*.ti,ab.	11
6	decision tree?.ti,ab.	0
7	flow chart?.ti,ab.	4
8	flow diagram???.ti,ab.	4
9	flowchart?.ti,ab.	1
10	((comply or complies or compliant or compliance) adj2 (policy or policies)).ti,ab.	0
11	((comply or complies or compliant or compliance) adj2 protocol*).ti,ab.	1
12	(best adj2 practi#e?).ti,ab.	31
13	(care adj (bundle or bundles)).ti,ab.	1
14	"care intervention?".ti,ab.	26
15	(care adj2 (path or paths or pathway or pathways)).ti,ab.	5
16	(care adj2 map*).ti,ab.	0

17	care model?.ti,ab.	11
18	(care adj2 plan*).ti,ab.	13
19	(clinical adj1 (path or paths or pathway or pathways)).ti,ab.	15
20	(clinical adj2 protocol?).ti,ab.	5
21	(comprehensive adj2 care).ti,ab.	6
22	(coordinated adj2 care).ti,ab.	0
23	(co-ordinated adj2 care).ti,ab.	1
24	(critical adj2 (path or paths or pathway or pathways)).ti,ab.	0
25	(decision adj2 tree?).ti,ab.	0
26	(decision? adj2 aid?).ti,ab.	17
27	(decision? adj2 analy*).ti,ab.	1
28	(decision? adj2 model*).ti,ab.	1
29	(decision? adj2 techni*).ti,ab.	0
30	((framework? or frame work?) and care).ti,ab.	26
31	((guideline or guidelines) adj2 care).ti,ab.	6
32	(guidance adj2 (introduc* or issu* or impact* or effect* or disseminat* or distribut* or implement*)).ti,ab.	2
33	(management adj2 protocol*).ti,ab.	6
34	(multicomponent adj2 (path? or pathway?)).ti,ab.	0
35	(multi-component adj2 (path? or pathway?)).ti,ab.	0
36	(multicomponent adj2 (program? or programme?)).ti,ab.	0
37	(multi-component adj2 (program? or programme?)).ti,ab.	5
38	(multimodal adj2 (path? or pathway?)).ti,ab.	0
39	(multimodal adj2 (program? or programme?)).ti,ab.	2
40	(multi-modal adj2 (program? or programme?)).ti,ab.	1
41	(nurs* adj2 protocol*).ti,ab.	0
42	(optimal* adj2 care).ti,ab.	4
43	(optimi* adj2 care).ti,ab.	1
44	optimi#ation.ti,ab.	16
45	(organi#ational adj1 model?).ti,ab.	1
46	((policy or policies) adj2 care).tw.	80
47	(practi#e adj1 parameter?).ti,ab.	0
48	(practi#e adj1 pattern?).tw.	101
49	(practi#e adj2 (protocol* or policy or policies or guideline*)).ti,ab.	76
50	(proactive* adj2 care).ti,ab.	1
51	(process?? adj2 (chart? or diagram* or flowchart*)).ti,ab.	0
52	((program or programme) adj project?).ti,ab.	0
53	((rule or rules) adj2 care).tw.	3
54	(standard? adj2 practi#e?).ti,ab.	34
55	surgical pathway?.ti,ab.	2
56	(treat* adj2 protocol?).ti,ab.	17
57	(treatment adj2 plan*).ti,ab.	23

58	(treatment* adj2 (path or paths or pathway or pathways)).ti,ab.	2
59	or/1-58 [Care pathway]	526
60	"older than 1##".ti,ab.	0
61	"older than 6#".ti,ab.	6
62	"older than 7#".ti,ab.	2
63	"older than 8#".ti,ab.	0
64	"older than 9#".ti,ab.	0
65	("over 1##" adj8 year?).ti,ab.	5
66	("over 6#" adj8 year?).ti,ab.	22
67	("over 7#" adj8 year?).ti,ab.	5
68	("over 8#" adj8 year?).ti,ab.	2
69	("over 9#" adj8 year?).ti,ab.	0
70	((old?? or advance?) adj (age or aging or ageing)).ti,ab.	15
71	((old?? or elder?? or senior?) adj (patient? or citizen?? or person? or people or geriatric* or population?)).ti,ab.	172
72	(aged adj2 "10# years").ti,ab.	0
73	(aged adj2 "6# years").ti,ab.	29
74	(aged adj2 "65 years").ti,ab.	18
75	(aged adj2 "7# years").ti,ab.	4
76	(aged adj2 "8# years").ti,ab.	1
77	(aged adj2 "9# years").ti,ab.	0
78	(elder* adj1 patient?).ti,ab.	14
79	(old adj age).ti,ab.	5
80	(old* adj1 patient?).ti,ab.	24
81	(older adult* or older client* or older patient* or older person* or older people).ti,ab.	155
82	centenarian*.ti,ab.	0
83	elder?.ti,ab.	2
84	elderly.ti,ab.	94
85	geriatri*.ti,ab.	20
86	grandfather*.ti,ab.	0
87	grandma??.ti,ab.	0
88	grandmother*.ti,ab.	0
89	grandpa??.ti,ab.	0
90	grandparent*.ti,ab.	0
91	nonagenarian*.ti,ab.	0
92	octagenarian*.ti,ab.	0
93	oncogeriatri*.ti,ab.	0
94	onco-geriatri*.ti,ab.	0
95	orthogeriatri*.ti,ab.	0
96	ortho-geriatri*.ti,ab.	0
97	psychogeriatri*.ti,ab.	2
98	psycho-geriatri*.ti,ab.	0

99	retiree*.ti,ab.	0
100	retirement?.ti,ab.	2
101	senior citizen*.ti,ab.	1
102	septuagenarian*.ti,ab.	0
103	sexagenarian*.ti,ab.	0
104	supercentenarian*.ti,ab.	0
105	super-centenarian*.ti,ab.	0
106	or/60-105 [Elderly]	289
107	59 and 106 [Care Pathway + Elderly]	23
108	(after adj6 (surgery or surgeries or surgical*)).ti,ab.	268
109	(before adj2 operat????).ti,ab.	5
110	(before adj3 procedur*).ti,ab.	12
111	(before adj6 (surgery or surgeries or surgical*)).ti,ab.	58
112	(during adj6 (surgery or surgeries or surgical*)).ti,ab.	114
113	(follow* adj6 (surgery or surgeries or surgical*)).ti,ab.	198
114	(operating adj2 room?).ti,ab.	12
115	(operating adj2 suite?).ti,ab.	1
116	(operating adj2 theater?).ti,ab.	0
117	(operating adj2 theatre?).ti,ab.	12
118	(operating adj2 unit?).ti,ab.	2
119	(prior adj3 operat????).ti,ab.	2
120	(prior adj3 surgery).ti,ab.	16
121	(prior adj3 procedur*).ti,ab.	6
122	(undergo* adj6 (surgery or surgeries or surgical*)).ti,ab.	297
123	an?esth*.ti,ab.	274
124	intraoperat*.ti,ab.	85
125	intra-operat*.ti,ab.	20
126	operation?.ti,ab.	149
127	operative*.ti,ab.	242
128	peri*procedur*.ti,ab.	4
129	perioperat*.ti,ab.	148
130	peri-operat*.ti,ab.	26
131	periprocedur*.ti,ab.	4
132	peri-procedur*.ti,ab.	3
133	peroperat*.ti,ab.	0
134	pos*ostomy.ti,ab.	0
135	pos*otomy.ti,ab.	1
136	post*ectomies.ti,ab.	0
137	post*ectomy.ti,ab.	3
138	post*otomies.ti,ab.	0
139	post*otomy.ti,ab.	1

140	post*surger*.ti,ab.	6
141	post-intervention*.ti,ab.	58
142	postoperat*.ti,ab.	479
143	post-operat*.ti,ab.	84
144	postproced*.ti,ab.	2
145	post-proced*.ti,ab.	7
146	postsurgical*.ti,ab.	10
147	post-surgical*.ti,ab.	22
148	preintervention*.ti,ab.	0
149	pre-intervention*.ti,ab.	5
150	preoperat*.ti,ab.	92
151	pre-operat*.ti,ab.	25
152	preprocedur*.ti,ab.	1
153	pre-procedur*.ti,ab.	2
154	reoperat*.ti,ab.	32
155	re-operat*.ti,ab.	20
156	re-resect*.ti,ab.	0
157	resect*.ti,ab.	189
158	or/108-157 [Perioperative & related terms]	1466
159	107 and 158 [Care Pathway + Elderly + Perioperative]	5
160	limit 159 to full systematic reviews	5

Ovid Emcare Nursing

Ovid Emcare Nursing 1995 to Present

#	Searches	Results
1	Algorithms/ and (patient care/ or collaborative care team/ or patient care planning/)	1345
2	Clinical Protocols/	35143
3	Critical Pathways/	3325
4	Decision Support Techniques/	9421
5	Decision Theory/	1699
6	exp Benchmarking/	1214
7	exp Decision Trees/	2662
8	exp Guidelines As Topic/	195037
9	exp Practice Guideline/	195037
10	exp Practice Guidelines As Topic/	195037
11	Guideline Adherence/	4869
12	Health planning guidelines/	35274
13	Models, Organizational/	4737
14	Patient Care Bundles/	410

15	Physician's Practice Patterns/	117944
16	"care intervention?".ti,ab.	3618
17	((comply or complies or compliant or compliance) adj2 (policy or policies)).ti,ab.	166
18	((comply or complies or compliant or compliance) adj2 protocol*).ti,ab.	388
19	((framework? or frame work?) and care).ti,ab.	24956
20	((guideline or guidelines) adj2 care).ti,ab.	2391
21	((policy or policies) adj2 care).tw.	3730
22	((program or programme) adj project?).ti,ab.	151
23	((rule or rules) adj2 care).tw.	61
24	((workflow* or work-flow*) adj2 map*4).mp.	30
25	((workflow* or work-flow*) adj2 process???).mp.	335
26	(best adj2 practi#e?).ti,ab.	15824
27	(care adj (bundle or bundles)).ti,ab.	454
28	(care adj2 (path or paths or pathway or pathways)).ti,ab.	3624
29	(care adj2 map*).ti,ab.	298
30	(care adj2 plan*).ti,ab.	11732
31	(clinical adj1 (path or paths or pathway or pathways)).ti,ab.	2026
32	(clinical adj2 protocol?).ti,ab.	3052
33	(comprehensive adj2 care).ti,ab.	4076
34	(coordinated adj2 care).ti,ab.	1140
35	(co-ordinated adj2 care).ti,ab.	81
36	(critical adj2 (path or paths or pathway or pathways)).ti,ab.	1200
37	(decision adj2 tree?).ti,ab.	3346
38	(decision? adj2 aid?).ti,ab.	2728
39	(decision? adj2 analy*).ti,ab.	5020
40	(decision? adj2 model*).ti,ab.	4588
41	(decision? adj2 techni*).ti,ab.	337
42	(guidance adj2 (introduc* or issu* or impact* or effect* or disseminat* or distribut* or implement*)).ti,ab.	932
43	(management adj2 protocol*).ti,ab.	2007
44	(multicomponent adj2 (path? or pathway?)).ti,ab.	4
45	(multi-component adj2 (path? or pathway?)).ti,ab.	4
46	(multicomponent adj2 (program? or programme?)).ti,ab.	250
47	(multi-component adj2 (program? or programme?)).ti,ab.	97
48	(multimodal adj2 (path? or pathway?)).ti,ab.	37
49	(multimodal adj2 (program? or programme?)).ti,ab.	356
50	(multi-modal adj2 (program? or programme?)).ti,ab.	51
51	(nurs* adj2 protocol*).ti,ab.	526
52	(optimal* adj2 care).ti,ab.	3546
53	(optimi* adj2 care).ti,ab.	2203
54	(organi#ational adj1 model?).ti,ab.	446
55	(practi#e adj1 parameter?).ti,ab.	511

56	(practi#e adj1 pattern?).tw.	3516
57	(practi#e adj2 (protocol* or policy or policies or guideline*)).ti,ab.	21383
58	(proactive* adj2 care).ti,ab.	292
59	(process?? adj2 (chart? or diagram* or flowchart* or map*4)).ti,ab.	1067
60	((rule or rules) and care).ti,ab.	5738
61	(standard? adj2 practi#e?).ti,ab.	5032
62	(treat* adj2 protocol?).ti,ab.	7558
63	(treatment adj2 plan*).ti,ab.	20403
64	(treatment* adj2 (path or paths or pathway or pathways)).ti,ab.	1244
65	algorhythm*.ti,ab.	19
66	algorism*.ti,ab.	22
67	(algorithm* and care).ti,ab.	7659
68	bench mark*.ti,ab.	135
69	benchmark*.ti,ab.	12975
70	care model?.ti,ab.	4580
71	decision tree?.ti,ab.	3285
72	flow chart?.ti,ab.	468
73	flow diagram???.ti,ab.	240
74	flowchart?.ti,ab.	513
75	gold standard?.ti,ab.	23512
76	optimi#ation.ti,ab.	25507
77	surgical pathway?.ti,ab.	59
78	algorithm/ [Embase]	56561
79	care bundle/ [Embase]	410
80	*clinical decision support system/ [Embase]	660
81	clinical pathway/ [Embase]	3325
82	clinical protocol/ [Embase]	35143
83	*decision support system/ [Embase]	4281
84	"decision tree"/ [Embase]	2662
85	*good clinical practice/ [Embase]	418
86	*health care planning/ [Embase]	5082
87	nursing care plan/ [Embase]	323
88	nursing protocol/ [Embase]	123
89	practice guideline/ [Embase]	149034
90	protocol compliance/ [Used for Guideline Adherence in Embase]	4869
91	or/1-90 [Algorithms or Pathways]	556614
92	"Aged, 80 and Over"/	214
93	Frail Elderly/	4542
94	exp Geriatrics/	11526
95	exp Gerontology/	2853
96	Geriatric Psychiatry/	2794

97	exp Health Services for the Aged/	29945
98	exp Geriatric Nursing/	2065
99	"older than 1##".ti,ab.	21
100	"older than 6#".ti,ab.	3195
101	"older than 7#".ti,ab.	1216
102	"older than 8#".ti,ab.	603
103	"older than 9#".ti,ab.	63
104	("over 1##" adj8 year?).ti,ab.	741
105	("over 6#" adj8 year?).ti,ab.	4196
106	("over 7#" adj8 year?).ti,ab.	1641
107	("over 8#" adj8 year?).ti,ab.	1018
108	("over 9#" adj8 year?).ti,ab.	346
109	((old?? or advance?) adj (age or aging or ageing)).ti,ab.	30156
110	((old?? or elder?? or senior?) adj (patient? or citizen?? or person? or people or geriatric* or population?)).ti,ab.	95153
111	(aged adj2 "10# years").ti,ab.	469
112	(aged adj2 "6# years").ti,ab.	21293
113	(aged adj2 "65 years").ti,ab.	10448
114	(aged adj2 "7# years").ti,ab.	10765
115	(aged adj2 "8# years").ti,ab.	5925
116	(aged adj2 "9# years").ti,ab.	2113
117	(elder* adj1 patient?).ti,ab.	26368
118	(old adj age).ti,ab.	9810
119	(old* adj1 patient?).ti,ab.	28928
120	(older adult* or older client* or older patient* or older person* or older people).ti,ab.	86293
121	centenarian*.ti,ab.	891
122	elder?.ti,ab.	9855
123	elderly.ti,ab.	100431
124	geriatri*.ti,ab.	29152
125	gerontol*.ti,ab.	7049
126	grandfather*.ti,ab.	299
127	grandma??.ti,ab.	72
128	grandmother*.ti,ab.	1042
129	grandpa??.ti,ab.	14
130	grandparent*.ti,ab.	1520
131	nonagenarian*.ti,ab.	604
132	octagenarian*.ti,ab.	10
133	oncogeriatri*.ti,ab.	54
134	onco-geriatri*.ti,ab.	17
135	orthogeriatri*.ti,ab.	244
136	ortho-geriatri*.ti,ab.	19
137	psychogeriatri*.ti,ab.	2270

138	psycho-geriatri*.ti,ab.	61
139	retiree*.ti,ab.	757
140	retirement?.ti,ab.	7111
141	senior citizen*.ti,ab.	816
142	septuagenarian*.ti,ab.	108
143	sexagenarian*.ti,ab.	38
144	supercentenarian*.ti,ab.	46
145	super-centenarian*.ti,ab.	1
146	aged/ or aged hospital patient/ or frail elderly/ or institutionalized elderly/ or very elderly/ [Embase]	744231
147	elderly care/ or exp geriatric care/ or home for the aged/ [Embase]	29789
148	geriatric care/ or geriatric hospital/ or geriatric nursing/ or geriatric patient/ or geriatric surgery/ or geriatrician/ or geriatrics/ [Embase]	30352
149	gerontologic nurse practitioner/ or gerontological research/ or gerontologist/ or gerontology/ [Embase]	3532
150	or/92-149 [Aged or Elderly or >=65 years of age]	849182
151	91 and 150 [Care Pathway + Elderly]	69059
152	anesthesist/	13376
153	exp *"Anesthesia and Analgesia"/	94754
154	exp Anesthesia Recovery Period/	3758
155	exp *Anesthesia/	49407
156	exp anesthesiologists/	1944
157	exp *Anesthesiology/	2346
158	exp *anesthetic agent/	59672
159	exp anesthetic recovery/	3758
160	exp *Anesthetics/	59672
161	exp Intraoperative care/	3326
162	exp Intraoperative Period/	49262
163	exp Perioperative Care/	18024
164	exp Perioperative Nursing/	1005
165	exp Perioperative Period/	18024
166	exp peroperative care/	3326
167	exp peroperative complication/	9020
168	exp Postoperative care/	24041
169	exp Postoperative Complications/	147082
170	exp Preoperative care/	8217
171	exp Specialties, Surgical/	1005961
172	exp surgeon/	41969
173	exp Surgeons/	41969
174	exp *surgery/	426072
175	exp *Surgical Procedures, Operative/	426072
176	Operating Rooms/	12655
177	Operative Time/	26816

178	Perioperative Nursing/	1005
179	Preanesthetic Medication/	4880
180	Preoperative Period/	17023
181	(after adj6 (surgery or surgeries or surgical*)).ti,ab.	93610
182	(before adj2 operat????).ti,ab.	3392
183	(before adj2 surgery).ti,ab.	14589
184	(before adj3 procedur*).ti,ab.	3761
185	(before adj3 procedur*).ti,ab.	3761
186	(before adj6 (surgery or surgeries or surgical*)).ti,ab.	24253
187	(during adj6 (surgery or surgeries or surgical*)).ti,ab.	33402
188	(follow* adj6 (surgery or surgeries or surgical*)).ti,ab.	35440
189	(operating adj2 room?).ti,ab.	11796
190	(operating adj2 suite?).ti,ab.	269
191	(operating adj2 theater?).ti,ab.	476
192	(operating adj2 theatre?).ti,ab.	1715
193	(operating adj2 unit?).ti,ab.	251
194	(prior adj3 operat????).ti,ab.	883
195	(prior adj3 procedur*).ti,ab.	1338
196	(prior adj3 surgery).ti,ab.	6014
197	(undergo* adj6 (surgery or surgeries or surgical*)).ti,ab.	36567
198	an?esth*.ti,ab.	123031
199	intraoperat*.ti,ab.	39906
200	intra-operat*.ti,ab.	4708
201	operation?.ti,ab.	84967
202	operative*.ti,ab.	72729
203	peri*procedur*.ti,ab.	2376
204	perioperat*.ti,ab.	34423
205	peri-operat*.ti,ab.	2356
206	periprocedur*.ti,ab.	2376
207	peri-procedur*.ti,ab.	357
208	peroperat*.ti,ab.	735
209	pos*ostomy.ti,ab.	58
210	pos*otomy.ti,ab.	690
211	post*ectomies.ti,ab.	1
212	post*ectomy.ti,ab.	2328
213	post*otomies.ti,ab.	0
214	post*otomy.ti,ab.	690
215	post*surger*.ti,ab.	2050
216	post-intervention*.ti,ab.	8800
217	postoperat*.ti,ab.	143646
218	post-operat*.ti,ab.	20380

219	postproced*.ti,ab.	3373
220	post-proced*.ti,ab.	2485
221	postsurgical*.ti,ab.	4918
222	post-surgical*.ti,ab.	2625
223	preintervention*.ti,ab.	2592
224	pre-intervention*.ti,ab.	2536
225	preoperat*.ti,ab.	78849
226	pre-operat*.ti,ab.	10082
227	preprocedur*.ti,ab.	1921
228	pre-procedur*.ti,ab.	1003
229	reoperat*.ti,ab.	7956
230	re-operat*.ti,ab.	1445
231	re-resect*.ti,ab.	110
232	reresect*.ti,ab.	28
233	resect*.ti,ab.	66926
234	or/152-233 [Perioperative]	1233763
235	151 and 234 [Care Pathway + Elderly + Perioperative]	17255
236	case series.mp,kw.	25521
237	cohort analysis/	161585
238	cohort*.mp,kw.	262145
239	Cross-Sectional Studies/	38464
240	cross-sectional study/	104926
241	double blind procedure/	48126
242	Double-Blind Method/	48126
243	doubleblind*.mp,kw.	360
244	Longitudinal Studies/	31284
245	Placebo*.mp,kw.	109794
246	placebo/	98601
247	Placebos/	98601
248	Prospective Studies/	87433
249	exp Randomized controlled trial/	176263
250	exp Randomized Controlled Trials as Topic/	63133
251	"randomized controlled trial (topic)"/ [embase]	63133
252	random*.mp,kw.	483379
253	controlled clinical trial/	59052
254	Controlled Clinical Trials As Topic/	3387
255	"controlled clinical trial (topic)"/	3387
256	meta analysis/	55489
257	meta-analysis as topic/	15393
258	"meta-analysis (topic)"/	15393
259	Pragmatic Clinical Trial/	252

260	Pragmatic Clinical Trials As Topic/	63133
261	systematic review/	102506
262	"systematic review (topic)"/	11903
263	validation study/	18794
264	evaluation study/	6959
265	exp case control study/	38539
266	exp Case-Control Studies/	38539
267	exp Cohort Studies/	161585
268	((single or double or treble or triple) adj3 (blind* or mask*)).mp,kw.	75854
269	(case control* adj2 (study or studies)).mp,kw.	45661
270	(controlled adj1 clinical adj2 (trial? or study or studies)).mp,kw.	71338
271	(cross-sectional* adj2 (study or studies)).mp,kw.	131052
272	(evaluation adj1 (study or studies)).mp,kw.	9986
273	(longitudinal* adj2 (study or studies)).mp,kw.	66727
274	(meta-anal* or metanal* or metaanal*).mp,kw.	92854
275	(overview? adj4 (review or reviews)).mp,kw.	3980
276	(pragmatic adj2 (trial? or study or studies)).mp,kw.	1414
277	(prospective* adj2 (study or studies)).mp,kw.	199803
278	(systematic adj4 (review or reviews or overview or overviews)).mp,kw.	123227
279	(validation adj1 (study or studies)).mp,kw.	20838
280	(content analys* or thematic analys* or narrative analys*).mp.	37255
281	(ethnol* or ethnog* or ethnonurs* or emic or etic).mp.	23021
282	(grounded adj5 theor*).mp,kw.	10625
283	(hermeneutic* or phenomenolog* or lived experience*).mp,kw.	19000
284	(meta-ethnog* or metaethnog* or meta-narrat* or metanarrat* or meta-interpret* or metainterpret*).mp,kw.	569
285	(metasynthes* or meta-synthes* or metasummar* or meta-summar* or metastud* or meta-stud*).mp,kw.	1021
286	action research.mp,kw.	3796
287	exp qualitative research/	48307
288	giorgi*.mp,kw.	411
289	nursing methodology research/	1588
290	qualitative.mp,kw.	133065
291	quasirandom*.mp.	84
292	or/236-291 [Studies]	1337325
293	235 and 292 [Care Pathway + Elderly + Perioperative + Studies]	7165
294	limit 293 to english language	7008
295	exp animals/ not (exp animals/ and exp humans/)	308047
296	294 not 295	7004
297	limit 294 to human	6868
298	296 or 297	7004
299	limit 298 to (embryo <first trimester> or infant <to one year> or child <unspecified age> or preschool child <1 to 6 years> or school child <7 to 12 years> or adolescent <13 to 17 years> or adult <18 to 64 years>)	4990

300	298 not 299	2014
301	limit 298 to aged <65+ years>	6237
302	300 or 301	6787
303	limit 302 to yr="2010 -Current"	4716
304	limit 303 to (book or book series or conference proceeding)	3
305	confer*.so.	18860
306	304 or 305	18862
307	303 not 306	4713
308	limit 307 to elderly	4636
309	medline.cr.	254778
310	308 not 309	4620
311	remove duplicates from 310	4603

CINAHL

#	Query	Limiters/Expanders	Last Run Via	Results
S12	S4 AND S7	Limiters- Published Date: 20100101-20201231; English Language; Peer Reviewed; Exclude MEDLINE records; Human; Age Groups: Aged: 65+ years, Aged, 80 and over<br>Expanders- Apply equivalent subjects<br>Search modes- Boolean/Phrase	Interface-EBSCOhost Research Databases<br>Search Screen-Advanced Search<br>Database-CINAHL with Full Text	2,493
S11	S4 AND S7	Limiters- Published Date: 20100101-20201231; English Language; Peer Reviewed; Exclude MEDLINE records; Human<br>Expanders- Apply equivalent subjects<br>Search modes- Boolean/Phrase	Interface-EBSCOhost Research Databases<br>Search Screen-Advanced Search<br>Database-CINAHL with Full Text	3,495
S10	S4 AND S7	Limiters- English Language; Peer Reviewed; Exclude MEDLINE records; Human<br>Expanders- Apply equivalent subjects<br>Search modes- Boolean/Phrase	Interface-EBSCOhost Research Databases<br>Search Screen-Advanced Search<br>Database-CINAHL with Full Text	3,893
S9	S4 AND S7	Limiters- English Language; Peer Reviewed; Human<br>Expanders- Apply equivalent subjects<br>Search modes- Boolean/Phrase	Interface-EBSCOhost Research Databases<br>Search Screen-Advanced Search<br>Database-CINAHL with Full Text	10,543
S8	S4 AND S7	Expanders- Apply equivalent subjects<br>Search modes- Boolean/Phrase	Interface-EBSCOhost Research Databases	12,769

			Search Screen- Advanced Search Database- CINAHL with Full Text	
S7	S5 OR S6	Expanders- Apply equivalent subjects Search modes- Boolean/Phrase	Interface- EBSCOhost Research Databases Search Screen- Advanced Search Database- CINAHL with Full Text	2,195,842
S6	((MH "Qualitative Research+") OR (MH "Evaluation Studies") OR (MH "Feasibility Studies") OR (MH "Interview") OR (MH "Patient Health Questionnaire") OR (MH "Grounded Theory") OR (MH "Hermeneutics") OR (MH "Nursing Methodology Research") OR (MH "Observational Study") OR (MH "Observational Studies as Topic") OR (MH "Patient Satisfaction") OR (MH "Pilot Projects") OR (MH "Program Evaluation") OR (MH "Surveys AND Questionnaires") OR ((discourse* OR discurs*) N3 analys*s) OR ((purpos* N4 sampl*) OR ("focus group*")) OR (account OR accounts OR unstructured OR open-ended OR "open ended" OR text* OR narrative*) OR ("action research" OR "cooperative inquir*" OR "co operativeinquir*" OR "co-operative inquir*") OR ("constant" AND (comparative OR comparison)) OR (corbin* N2 strauss*) OR (emic OR etic OR hermeneutic* OR heuristic* OR semiotic*) OR (data N1 saturat*) OR "participant observ*" OR (evaluat* N2 (study OR studies)) OR ("field" AND (study OR studies OR research)) OR ("grounded" AND (theor* OR study OR studies OR research OR analys*s)) OR (humanistic OR existential OR experiential OR paradigm*) OR ("life stor*" OR "women* stor*") OR ("life world" OR life-world OR "conversation analys") OR ((lived OR life) "adj experience*") OR ("merleauponty*") OR ("observational" AND (study OR studies OR research)) OR ("social construct*" OR (postmodern* OR "post- structural*") OR ("post structural*" OR poststructural*) OR "post modern*" OR post-modern* OR feminis* OR interpret*) OR (survey# OR surveyed OR surveying) OR (theme* OR thematic) OR ("van kaam*") OR ("van manen*") OR "biographical method#" OR "cluster sampl*" OR colaizzi* OR "content analys" OR ethnograph* OR "ethnological research" OR ethnonursing OR foucault* OR glaser* OR heidegger* OR "human science" OR "narrative analys" OR "observational method*" OR phenomenol* OR qualitative OR questionnaire* OR spiegelberg* OR "theoretical sampl*" OR (client* N2 (satisfaction OR satisfied OR satisfy*)) OR ("content analys*" OR "thematic analys*" OR "narrative analys*") OR (ethnol* OR ethnog* OR ethnonurs* OR emic OR etic) OR (feasib* N2 (study OR studies)) OR (Grounded N5 theor*) OR (hermeneutic* OR phenomenolog* OR "lived experience*") OR (integrat* N1 model#) OR (meta-ethnog* OR metaethnog* OR meta-narrat* OR metanarrat* OR meta-interpret* OR metainterpret*) OR (metasynthes* OR meta-synthes* OR metasummar* OR meta-summar* OR metastud* OR meta-stud*) OR (multiple N1 perspective#) OR (patient## N2 (satisfaction OR satisfied OR satisfy*)) OR (personal N1 (story OR stories)) OR (personal N1 account#**) OR (program* N3 evaluat*) OR (qualitative N5 metaanaly*) OR (qualitative N5 meta-analy*) OR (therapeutic N1 model*) OR (treatment* N1 model*) OR TI "action research" OR AB "action research" OR contextual* OR "focus group*" OR "frame	Expanders- Apply equivalent subjects Search modes- Boolean/Phrase	Interface- EBSCOhost Research Databases Search Screen- Advanced Search Database- CINAHL with Full Text	1,390,287

	work*" OR framework* OR giorgi* OR interview* OR "multimethod inquir*" OR "multi-method inquir*" OR multiperspective* OR multi-perspective* OR narrative* OR phenomenological* OR qualitative* OR qualitative OR questionnaire* OR thematic OR theme OR themes)			
S5	(PT "Clinical Trial, Phase III" OR (MH "Clinical Trial, Phase III") OR PT "Clinical Trial" OR (MH "Clinical Trials, Phase III as Topic") OR PT "Comparative Study" OR (MH "Comparative Study") OR PT "Controlled Clinical Trial" OR (MH "Controlled Clinical Trial") OR (MH "Controlled Clinical Trials as Topic") OR (MH "Cross-Sectional Studies") OR (MH "Double-Blind Method") OR PT "Equivalence Trial" OR (MH "Equivalence Trial") OR (MH "Equivalence Trials as Topic") OR PT "Evaluation Studies" OR (MH "Case-Control Studies+") OR (MH "Cohort Studies+") OR (MH "Randomized Controlled Trial+") OR (MH "Randomized Controlled Trials as Topic+") OR (MH "Longitudinal Studies") OR (MH "Meta-Analysis as Topic") OR (MH "Meta-Analysis") OR (MH "Multicenter Studies as Topic") OR PT "Multicenter Study" OR (MH "Multicenter Study") OR (MH "Placebos") OR PT "Practice Guideline" OR PT "Pragmatic Clinical Trial" OR (MH "Pragmatic Clinical Trial") OR (MH "Pragmatic Clinical Trials as Topic") OR (MH "Prospective Studies") OR PT "Randomized Controlled Trial" OR (MH "Retrospective Studies") OR (MH "Systematic Review") OR PT "Systematic Review" OR (MH "Systematic Reviews as Topic") OR (MH "Validation Studies") OR PT "Validation Studies" OR ("phase 3" OR phase3 OR "phase III") OR ((multicenter* OR multicentre*) N2 (trial* OR study OR studies)) OR ((noninferiority OR non-inferiority) N4 (trial* OR study OR studies)) OR ((single OR double OR triple OR treble) N3 (blind* OR mask*)) OR ("case control*" N2 (study OR studies)) OR (comparative N2 (trial* OR study OR studies)) OR (conceal* N2 allocat*) OR (controlled N1 clinical N2 (trial* OR study OR studies)) OR (cross-sectional* N2 (study OR studies)) OR (equivalen* N4 (trial* OR study OR studies)) OR (evaluation N1 (study OR studies)) OR (longitudinal* N2 (study OR studies)) OR (meta-anal* OR metanal* OR metaanal*) OR (overview* N4 (review OR reviews)) OR (pragmatic N2 (trial* OR study OR studies)) OR (prospective* N2 (study OR studies)) OR (retrospective* N2 (study OR studies)) OR (superiority N4 (trial* OR study OR studies)) OR (systematic N4 (review OR reviews OR overview OR overviews)) OR (validation N1 (study OR studies)) OR cohort* OR placebo* OR quasirandom* OR random*)	Expanders- Apply equivalent subjects<br>Search modes- Boolean/Phrase	Interface- EBSCOhost Research Databases<br>Search Screen- Advanced Search<br>Database- CINAHL with Full Text	1,372,757
S4	S1 AND S2 AND S3	Expanders- Apply equivalent subjects<br>Search modes- Boolean/Phrase	Interface- EBSCOhost Research Databases<br>Search Screen- Advanced Search<br>Database- CINAHL with Full Text	15,950
S3	((MH "Anesthesia AND Analgesia+") OR (MH "Anesthesia Recovery Period+") OR (MH "Anesthesia+") OR (MH "anesthesiologists+") OR (MH "Anesthesiology+") OR (MH "Anesthetics+") OR (MH "Intraoperative care+") OR (MH "Intraoperative Period+") OR (MH "Perioperative Care+") OR (MH "Perioperative Nursing+") OR (MH "Perioperative Period+") OR (MH "Postoperative care+") OR (MH "Postoperative Complications+") OR (MH "Preoperative care+") OR (MH "Specialties, Surgical+") OR (MH "Surgeons+") OR (MH "Surgical Procedures, Operative+") OR (MH "Operating Rooms") OR (MH "Operative Time") OR (MH "Perioperative Nursing") OR (MH "Preanesthetic Medication") OR (MH "Preoperative Period") OR (after	Expanders- Apply equivalent subjects<br>Search modes- Boolean/Phrase	Interface- EBSCOhost Research Databases<br>Search Screen- Advanced Search<br>Database- CINAHL with Full Text	2,859,809

	N6 (surgery OR surgeries OR surgical*)) OR (before N2 operat*) OR (before N3 procedur*) OR (before N6 (surgery OR surgeries OR surgical*)) OR (during N6 (surgery OR surgeries OR surgical*)) OR (follow* N6 (surgery OR surgeries OR surgical*)) OR (operating N2 room*) OR (operating N2 suite*) OR (operating N2 theater*) OR (operating N2 theatre*) OR (operating N2 unit*) OR (prior N3 operat*) OR (prior N3 surgery) OR (prior N3 procedur*) OR (undergo* N6 (surgery OR surgeries OR surgical*)) OR an*esth* OR intraoperat* OR intra-operat* OR operation* OR operative* OR peri*procedur* OR perioperat* OR peri-operat* OR periprocedur* OR peri-procedur* OR peroperat* OR pos*ostomy OR pos*otomy OR post*ectomies OR post*ectomy OR post*otomies OR post*otomy OR post*surger* OR post-intervention* OR postoperat* OR post-operat* OR postproced* OR post-proced* OR postsurgical* OR post-surgical* OR preintervention* OR pre-intervention* OR preoperat* OR pre-operat* OR preprocedur* OR pre-procedur* OR reoperat* OR re-operat* OR re-resect* OR resect*)			
S2	((MH "aged+") OR (MH "aged, 80 AND over") OR (MH "frail elderly") OR (MH "Geriatrics+") OR (MH "Geriatric Assessment+") OR (MH "Geriatric Psychiatry") OR (MH "Health Services for the Aged+") OR (MH "Geriatric Nursing+") OR "older than 1*" OR "older than 6*" OR "older than 7*" OR "older than 8*" OR "older than 9*" OR ("over 1*" N8 year*) OR ("over 6*" N8 year*) OR ("over 7*" N8 year*) OR ("over 8*" N8 year*) OR ("over 9*" N8 year*) OR ((old* OR advance*) AND (age OR aging OR ageing)) OR ((old* OR elder* OR senior*) AND (patient* OR citizen* OR person* OR people OR geriatric* OR population*)) OR (aged N2 "10* years") OR (aged N2 "6* years") OR (aged N2 "65 years") OR (aged N2 "7* years") OR (aged N2 "8* years") OR (aged N2 "9* years") OR (elder* N1 patient*) OR ("old age") OR (old* N1 patient*) OR ("older adult*" OR "older client*" OR "older patient*" OR "older person*" OR "older people") OR centenarian* OR elder* OR elderly OR geriatri* OR grandfather* OR grandma* OR grandmother* OR grandpa* OR grandparent* OR nonagenarian* OR octagenarian* OR oncogeriatri* OR onco-geriatri* OR orthogeriatri* OR ortho-geriatri* OR psychogeriatri* OR psycho-geriatri* OR retiree* OR retirement* OR "senior citizen*" OR septuagenarian* OR sexagenarian* OR supercentenarian* OR super-centenarian*)	Expanders- Apply equivalent subjects<br>Search modes- Boolean/Phrase	Interface- EBSCOhost Research Databases<br>Search Screen- Advanced Search<br>Database- CINAHL with Full Text	1,021,193
S1	((MH "Algorithms") AND ((MH "Patient Care") OR (MH "Progressive Patient Care") OR (MH "Patient Care Planning") OR (MH "Patient Care Management") OR (MH "Episode of Care")) OR (MH "Clinical Protocols") OR (MH "Continuity of Patient Care") OR (MH "Critical Pathways") OR (MH "Decision Support Techniques") OR (MH "Decision Theory") OR (MH "Benchmarking+") OR (MH "Decision Trees+") OR (MH "Guideline+") OR (MH "Guidelines As Topic+") OR (MH "Practice Guideline+") OR (MH "Practice Guidelines As Topic+") OR (MH "Guideline Adherence") OR (MH "Health planning guidelines") OR (MH "Models, Organizational") OR (MH "Patient Care Bundles") OR (MH "Physician's Practice Patterns") OR algorhythm* OR algorism* OR (algorithm* AND care) OR "bench mark*" OR benchmark* OR "decision tree*" OR "flow chart*" OR "flow diagram*" OR flowchart* OR ((comply OR complies OR compliant OR compliance) N2 (policy OR policies)) OR ((comply OR complies OR compliant OR compliance) N2 protocol*) OR (best N2 practi*e*) OR ("care" AND (bundle OR bundles)) OR "care intervention*" OR (care N2 (path OR paths OR pathway OR pathways)) OR (care N2 map*) OR "care model*" OR (care N2 plan*) OR (clinical N1 (path OR paths OR pathway OR pathways)) OR (clinical N2 protocol*) OR (comprehensive N2 care) OR (coordinated N2 care) OR (co-ordinated N2 care) OR	Expanders- Apply equivalent subjects<br>Search modes- Boolean/Phrase	Interface- EBSCOhost Research Databases<br>Search Screen- Advanced Search<br>Database- CINAHL with Full Text	110,962

	(critical N2 (path OR paths OR pathway OR pathways)) OR (decision N2 tree*) OR (decision* N2 aid*) OR (decision* N2 analy*) OR (decision* N2 model*) OR (decision* N2 techni*) OR ((framework* OR "frame work*") AND care) OR ((guideline OR guidelines) N2 care) OR (guidance N2 (introduc* OR issu* OR impact* OR effect* OR disseminat* OR distribut* OR implement*)) OR (management N2 protocol*) OR (multicomponent N2 (path* OR pathway*)) OR (multi-component N2 (path* OR pathway*)) OR (multicomponent N2 (program* OR programme*)) OR (multi-component N2 (program* OR programme*)) OR (multimodal N2 (path* OR pathway*)) OR (multimodal N2 (program* OR programme*)) OR (multi-modal N2 (program* OR programme*)) OR (nurs* N2 protocol*) OR (optimal* N2 care) OR (optimi* N2 care) OR optimi*ation OR (organi*ational N1 model*) OR ((policy OR policies) N2 care) OR (practi*e N1 parameter*) OR (practi*e N1 pattern*) OR (practi*e N2 (protocol* OR policy OR policies OR guideline*)) OR (proactive* N2 care) OR (process* N2 (chart* OR diagram* OR flowchart*)) OR ((program OR programme) AND project*) OR ((rule OR rules) N2 care) OR (standard* N2 practi*e*) OR "surgical pathway*" OR (treat* N2 protocol*) OR (treatment N2 plan*) OR (treatment* N2 (path OR paths OR pathway OR pathways))) AND ((MH "Patient Care+") OR (MH "Patient Care Plans+") OR (MH "Patient Centered Care") OR (MH "Multidisciplinary Care Team+") OR (MH "Continuity of Patient Care+") OR (MH "Total Patient Care Nursing") OR (MH "Progressive Patient Care") OR (MH "Quality Patient Care Scale"))			

Web of Science

Web of Science Core Collection: Citation Indexes

- [x] Science Citation Index Expanded (SCI-EXPANDED) --1900-present
- [x] Social Sciences Citation Index (SSCI) --1900-present
- [x] Arts & Humanities Citation Index (A&HCI) --1975-present
- [ ] Conference Proceedings Citation Index- Science (CPCI-S) --1990-present
- [ ] Conference Proceedings Citation Index- Social Science & Humanities (CPCI-SSH) --1990-present
- [ ] Book Citation Index– Science (BKCI-S) --2005-present
- [ ] Book Citation Index– Social Sciences & Humanities (BKCI-SSH) --2005-present
- [x] Emerging Sources Citation Index (ESCI) --2005-present

Data last updated: 2020-01-22

# 10	287	(#8 NOT #9) *AND* **LANGUAGE:** (English) *AND***DOCUMENT TYPES:** (Article)<br>Indexes=SCI-EXPANDED, SSCI, A&HCI, ESCI Timespan=2010-2020
# 9	22,455,084	PMID=(0* OR 1* OR 2* OR 3* OR 4* OR 5* OR 6* OR 7* OR 8* OR 9*)<br>Indexes=SCI-EXPANDED, SSCI, A&HCI, ESCI Timespan=1900-2020
# 8	3,009	#7 AND #4<br>Indexes=SCI-EXPANDED, SSCI, A&HCI, ESCI Timespan=2010-2020
# 7	5,148,367	#6 OR #5<br>Indexes=SCI-EXPANDED, SSCI, A&HCI, ESCI Timespan=2010-2020
# 6	3,930,224	(TS=("Qualitative Research" OR "Evaluation Studies" OR "Feasibility Studies" OR Interview OR "Patient Health

		Questionnaire" OR "Grounded Theory" OR Hermeneutics OR "Nursing Methodology Research" OR "Observational Study" OR "Observational Studies" OR "Patient Satisfaction" OR "Pilot Projects" OR "Program Evaluation" OR "Surveys AND Questionnaires" OR ((discourse* OR discurs*) NEAR/3 analys*s) OR ((purpos* NEAR/4 sampl*) OR ("focus group*")) OR (account OR accounts OR unstructured OR open-ended OR "open ended" OR text* OR narrative*) OR ("action research" OR "cooperative inquir*" OR "co operativeinquir*" OR "co- operative inquir*") OR ("constant" AND (comparative OR comparison)) OR (corbin* NEAR/2 strauss*) OR (emic OR etic OR hermeneutic* OR heuristic* OR semiotic*) OR (data NEAR/1 saturat*) OR "participant observ*" OR (evaluat* NEAR/2 (study OR studies)) OR ("field" AND (study OR studies OR research)) OR ("grounded" AND (theor* OR study OR studies OR research OR analys*s)) OR (humanistic OR existential OR experiential OR paradigm*) OR ("life stor*" OR "women* stor*") OR ("life world" OR life-world OR "conversation analys") OR ((lived OR life) "adj experience*") OR ("merleauponty*") OR ("observational" AND (study OR studies OR research)) OR ("social construct*" OR (postmodern* OR "post- structural*") OR ("post structural*" OR poststructural*) OR "post modern*" OR post-modern* OR feminis* OR interpret*) OR (survey* OR surveyed OR surveying) OR (theme* OR thematic) OR ("van kaam*") OR ("van manen*") OR "biographical method*" OR "cluster sampl*" OR colaizzi* OR "content analys" OR ethnograph* OR "ethnological research" OR ethnonursing OR foucault* OR glaser* OR heidegger* OR "human science" OR "narrative analys" OR "observational method*" OR phenomenol* OR qualitative OR questionnaire* OR spiegelberg* OR "theoretical sampl*" OR (client* NEAR/2 (satisfaction OR satisfied OR satisfy*)) OR ("content analys*" OR "thematic analys*" OR "narrative analys*") OR (ethnol* OR ethnog* OR ethnonurs* OR emic OR etic) OR (feasib* NEAR/2 (study OR studies)) OR (Grounded NEAR/5 theor*) OR (hermeneutic* OR phenomenolog* OR "lived experience*") OR (integrat* NEAR/1 model*) OR (meta-ethnog* OR metaethnog* OR meta-narrat* OR metanarrat* OR meta-interpret* OR metainterpret*) OR (metasynthes* OR meta-synthes* OR metasummar* OR meta-summar* OR metastud* OR meta-stud*) OR (multiple NEAR/1 perspective*) OR (patient** NEAR/2 (satisfaction OR satisfied OR satisfy*)) OR (personal NEAR/1 (story OR stories)) OR (personal NEAR/1 account***) OR (program* NEAR/3 evaluat*) OR (qualitative NEAR/5 metaanaly*) OR (qualitative NEAR/5 meta-analy*) OR (therapeutic NEAR/1 model*) OR (treatment* NEAR/1 model*) OR "action research" OR contextual* OR "focus group*" OR "frame work*" OR framework* OR giorgi* OR interview* OR "multimethod inquir*" OR "multi-method inquir*" OR multiperspective* OR multi-perspective* OR narrative* OR phenomenological* OR qualitative* OR qualitative OR questionnaire* OR thematic OR theme OR themes)) *AND* **LANGUAGE:** (English) *AND***DOCUMENT TYPES:** (Article)<br>Indexes=SCI-EXPANDED, SSCI, A&HCI, ESCI Timespan=2010-2020
# 5	1,861,082	(TS=("Clinical Trial" OR "Clinical Trials" OR "Comparative Study" OR "Controlled Clinical Trial" OR "Controlled Clinical Trials" OR "Cross-Sectional Studies" OR "Double-Blind Method" OR "Equivalence Trial" OR "Equivalence Trials" OR "Evaluation Studies" OR "Case-Control Studies" OR "Cohort Studies" OR "Randomized Controlled Trial" OR "Randomized Controlled Trials" OR "Longitudinal Studies" OR "Meta-Analysis as Topic" OR Meta-Analysis OR "Multicenter Studies" OR "Multicenter Study" OR Placebos OR "Practice Guideline" OR "Pragmatic Clinical Trial" OR "Pragmatic Clinical Trials" OR "Prospective Studies" OR "Randomized Controlled Trial" OR "Retrospective Studies" OR "Systematic Review" OR "Systematic Reviews" OR "Validation Studies" OR "Validation Study" OR ("phase 3" OR phase3 OR "phase III") OR ((multicenter* OR multicentre*) NEAR/2 (trial* OR study OR studies)) OR ((noninferiority OR non-inferiority) NEAR/4 (trial* OR study OR studies)) OR ((single OR double OR triple OR treble) NEAR/3 (blind* OR mask*)) OR ("case control*" NEAR/2 (study OR studies)) OR (comparative NEAR/2 (trial* OR study OR studies)) OR (conceal* NEAR/2 allocat*) OR (controlled NEAR/1 clinical NEAR/2 (trial* OR study OR studies)) OR (cross-sectional* NEAR/2 (study OR studies)) OR (equivalen* NEAR/4 (trial* OR study OR studies)) OR (evaluation NEAR/1 (study OR studies)) OR (longitudinal* NEAR/2 (study OR studies)) OR (meta-anal* OR metanal* OR metaanal*) OR (overview* NEAR/4 (review OR reviews)) OR (pragmatic NEAR/2 (trial* OR study OR studies)) OR (prospective* NEAR/2 (study OR studies)) OR (retrospective* NEAR/2 (study OR studies)) OR (superiority NEAR/4 (trial* OR study OR studies)) OR (systematic NEAR/4 (review OR reviews OR overview OR overviews)) OR (validation NEAR/1 (study OR studies)) OR cohort* OR placebo* OR quasirandom* OR random*)) *AND* **LANGUAGE:** (English) *AND***DOCUMENT TYPES:** (Article)<br>Indexes=SCI-EXPANDED, SSCI, A&HCI, ESCI Timespan=2010-2020
# 4	6,660	#3 AND #2 AND #1<br>Indexes=SCI-EXPANDED, SSCI, A&HCI, ESCI Timespan=All years
# 3	2,050,200	(TS=("Anesthesia AND Analgesia" OR "Anesthesia Recovery Period" OR Anesthesia OR anesthesiologists OR Anesthesiology OR Anesthetics OR "Intraoperative care" OR "Intraoperative Period" OR "Perioperative Care" OR "Perioperative Nursing" OR "Perioperative Period" OR "Postoperative care" OR "Postoperative Complications" OR "Preoperative care" OR "Specialties, Surgical" OR Surgeons OR "Surgical Procedures, Operative" OR "Operating Rooms" OR "Operative Time" OR "Perioperative Nursing" OR "Preanesthetic Medication" OR "Preoperative Period" OR (after NEAR/6 (surgery OR surgeries OR surgical*)) OR (before NEAR/2 operat*) OR (before NEAR/3 procedur*) OR (before NEAR/6 (surgery OR surgeries OR surgical*)) OR (during NEAR/6 (surgery

		OR surgeries OR surgical*)) OR (follow* NEAR/6 (surgery OR surgeries OR surgical*)) OR (operating NEAR/2 room*) OR (operating NEAR/2 suite*) OR (operating NEAR/2 theater*) OR (operating NEAR/2 theatre*) OR (operating NEAR/2 unit*) OR (prior NEAR/3 operat*) OR (prior NEAR/3 surgery) OR (prior NEAR/3 procedur*) OR (undergo* NEAR/6 (surgery OR surgeries OR surgical*)) OR an*esth* OR intraoperat* OR intra-operat* OR operation* OR operative* OR peri*procedur* OR perioperat* OR peri-operat* OR periprocedur* OR peri-procedur* OR peroperat* OR pos*ostomy OR pos*otomy OR post*ectomies OR post*ectomy OR post*otomies OR post*otomy OR post*surger* OR post-intervention* OR postoperat* OR post-operat* OR postproced* OR post-proced* OR postsurgical* OR post-surgical* OR preintervention* OR pre-intervention* OR preoperat* OR pre-operat* OR preprocedur* OR pre-procedur* OR reoperat* OR re-operat* OR re-resect* OR resect*)) *AND* **LANGUAGE:** (English) *AND***DOCUMENT TYPES:** (Article)<br>Indexes=SCI-EXPANDED, SSCI, A&HCI, ESCI Timespan=All years
# 2	1,254,765	(TS=("elderly" OR "frail elderly" OR Geriatrics OR "Geriatric Assessment" OR "Geriatric Psychiatry" OR "Health Services for the Aged" OR "Geriatric Nursing" OR "older than 1*" OR "older than 6*" OR "older than 7*" OR "older than 8*" OR "older than 9*" OR ("over 1*" NEAR/8 year*) OR ("over 6*" NEAR/8 year*) OR ("over 7*" NEAR/8 year*) OR ("over 8*" NEAR/8 year*) OR ("over 9*" NEAR/8 year*) OR ((old* OR advance*) AND (age OR aging OR ageing)) OR ((old* OR elder* OR senior*) AND (patient* OR citizen* OR person* OR people OR geriatric* OR population*)) OR (aged NEAR/2 "10* years") OR (aged NEAR/2 "6* years") OR (aged NEAR/2 "65 years") OR (aged NEAR/2 "7* years") OR (aged NEAR/2 "8* years") OR (aged NEAR/2 "9* years") OR (elder* NEAR/1 patient*) OR ("old age") OR (old* NEAR/1 patient*) OR ("older adult*" OR "older client*" OR "older patient*" OR "older person*" OR "older people") OR centenarian* OR elder* OR elderly OR geriatri* OR grandfather* OR grandma* OR grandmother* OR grandpa* OR grandparent* OR nonagenarian* OR octagenarian* OR oncogeriatri* OR onco-geriatri* OR orthogeriatri* OR ortho-geriatri* OR psychogeriatri* OR psycho-geriatri* OR retiree* OR retirement* OR "senior citizen*" OR septuagenarian* OR sexagenarian* OR supercentenarian* OR super-centenarian*)) *AND* **LANGUAGE:** (English) *AND***DOCUMENT TYPES:** (Article)<br>Indexes=SCI-EXPANDED, SSCI, A&HCI, ESCI Timespan=All years
# 1	1,197,397	(TS=(algorhythm* OR algorism* OR (algorithm* AND care) OR "bench mark*" OR benchmark* OR "decision tree*" OR "flow chart*" OR "flow diagram*" OR flowchart* OR ((comply OR complies OR compliant OR compliance) NEAR/2 (policy OR policies)) OR ((comply OR complies OR compliant OR compliance) NEAR/2 protocol*) OR (best NEAR/2 practi*e*) OR ("care" AND (bundle OR bundles)) OR "care intervention*" OR (care NEAR/2 (path OR paths OR pathway OR pathways)) OR (care NEAR/2 map*) OR "care model*" OR (care NEAR/2 plan*) OR (clinical NEAR/1 (path OR paths OR pathway OR pathways)) OR (clinical NEAR/2 protocol*) OR (comprehensive NEAR/2 care) OR (coordinated NEAR/2 care) OR (co-ordinated NEAR/2 care) OR (critical NEAR/2 (path OR paths OR pathway OR pathways)) OR (decision NEAR/2 tree*) OR (decision* NEAR/2 aid*) OR (decision* NEAR/2 analy*) OR (decision* NEAR/2 model*) OR (decision* NEAR/2 techni*) OR ((framework* OR "frame work*") AND care) OR ((guideline OR guidelines) NEAR/2 care) OR (guidance NEAR/2 (introduc* OR issu* OR impact* OR effect* OR disseminat* OR distribut* OR implement*)) OR (management NEAR/2 protocol*) OR (multicomponent NEAR/2 (path* OR pathway*)) OR (multi-component NEAR/2 (path* OR pathway*)) OR (multicomponent NEAR/2 (program* OR programme*)) OR (multi-component NEAR/2 (program* OR programme*)) OR (multimodal NEAR/2 (path* OR pathway*)) OR (multimodal NEAR/2 (program* OR programme*)) OR (multi-modal NEAR/2 (program* OR programme*)) OR (nurs* NEAR/2 protocol*) OR (optimal* NEAR/2 care) OR (optimi* NEAR/2 care) OR optimi*ation OR (organi*ational NEAR/1 model*) OR ((policy OR policies) NEAR/2 care) OR (practi*e NEAR/1 parameter*) OR (practi*e NEAR/1 pattern*) OR (practi*e NEAR/2 (protocol* OR policy OR policies OR guideline*)) OR (proactive* NEAR/2 care) OR (process* NEAR/2 (chart* OR diagram* OR flowchart*)) OR ((program OR programme) "adj project*") OR ((rule OR rules) NEAR/2 care) OR (standard* NEAR/2 practi*e*) OR "surgical pathway*" OR (treat* NEAR/2 protocol*) OR (treatment NEAR/2 plan*) OR (treatment* NEAR/2 (path OR paths OR pathway OR pathways)))) *AND* **LANGUAGE:** (English) *AND***DOCUMENT TYPES:** (Article)<br>Indexes=SCI-EXPANDED, SSCI, A&HCI, ESCI Timespan=All years

Scopus

294 document results

(((INDEXTERMS (*"Algorithms"*) AND (INDEXTERMS (*"Patient Care"*) OR INDEXTERMS (*"Progressive Patient Care"*) OR INDEXTERMS (*"Patient Care Planning"*) OR INDEXTERMS (*"Patient Care Management"*) OR INDEXTERMS (*"Episode of Care"*)) OR INDEXTERMS (*"Clinical Protocols"*) OR INDEXTERMS (*"Continuity of Patient Care"*) OR INDEXTERMS (*"Critical Pathways"*)

OR INDEXTERMS (*"Decision Support Techniques"*) OR INDEXTERMS (*"Decision Theory"*) OR INDEXTERMS (*"Benchmarking"*) OR INDEXTERMS (*"Decision Trees"*) OR INDEXTERMS (*"Guideline"*) OR INDEXTERMS (*"Guidelines As Topic"*) OR INDEXTERMS (*"Practice Guideline"*) OR INDEXTERMS (*"Practice Guidelines As Topic"*) OR INDEXTERMS (*"Guideline Adherence"*) OR INDEXTERMS (*"Health planning guidelines"*) OR INDEXTERMS (*"Models, Organizational"*) OR INDEXTERMS (*"Patient Care Bundles"*) OR INDEXTERMS (*"Physician's Practice Patterns"*) OR TITLE-ABS-KEY (*"algorhythm*"*) OR TITLE-ABS-KEY (*"algorism*"*) OR (*"algorithm*"* AND *"care"*) OR TITLE-ABS-KEY (*"bench mark*"*) OR TITLE-ABS-KEY (*"benchmark*"*) OR TITLE-ABS-KEY (*"decision tree*"*) OR TITLE-ABS-KEY (*"flow chart*"*) OR TITLE-ABS-KEY (*"flow diagram*"*) OR TITLE-ABS-KEY (*"flowchart*"*) OR ((*"comply"* OR *"complies"* OR *"compliant"* OR *"compliance"*) W/2 (*"policy"* OR *"policies"*)) OR ((*"comply"* OR *"complies"* OR *"compliant"* OR *"compliance"*) W/2 *"protocol*"*) OR (*"best"* W/2 *"practi*e*"*) OR (*"care"* AND (*"bundle"* OR *"bundles"*)) OR TITLE-ABS-KEY (*"care intervention*"*) OR (*"care"* W/2 (*"path"* OR *"paths"* OR *"pathway"* OR *"pathways"*)) OR (*"care"* W/2 *"map*"*) OR TITLE-ABS-KEY (*"care model*"*) OR (*"care"* W/2 *"plan*"*) OR (*"clinical"* W/1 (*"path"* OR *"paths"* OR *"pathway"* OR *"pathways"*)) OR (*"clinical"* W/2 *"protocol*"*) OR (*"comprehensive"* W/2 *"care"*) OR (*"coordinated"* W/2 *"care"*) OR (*"co-ordinated"* W/2 *"care"*) OR (*"critical"* W/2 (*"path"* OR *"paths"* OR *"pathway"* OR *"pathways"*)) OR (*"decision"* W/2 *"tree*"*) OR (*"decision*"* W/2 *"aid*"*) OR (*"decision*"* W/2 *"analy*"*) OR (*"decision*"* W/2 *"model*"*) OR (*"decision*"* W/2 *"techni*"*) OR ((*"framework*"* OR *"frame work*"*) AND *"care"*) OR ((*"guideline"* OR *"guidelines"*) W/2 *"care"*) OR (*"guidance"* W/2 (*"introduc*"* OR *"issu*"* OR *"impact*"* OR *"effect*"* OR *"disseminat*"* OR *"distribut*"* OR *"implement*"*)) OR (*"management"* W/2 *"protocol*"*) OR (*"multicomponent"* W/2 (*"path*"* OR *"pathway*"*)) OR (*"multi-component"* W/2 (*"path*"* OR *"pathway*"*)) OR (*"multicomponent"* W/2 (*"program*"* OR *"programme*"*)) OR (*"multi-component"* W/2 (*"program*"* OR *"programme*"*)) OR (*"multimodal"* W/2 (*"path*"* OR *"pathway*"*)) OR (*"multimodal"* W/2 (*"program*"* OR *"programme*"*)) OR (*"multi-modal"* W/2 (*"program*"* OR *"programme*"*)) OR (*"nurs*"* W/2 *"protocol*"*) OR (*"optimal*"* W/2 *"care"*) OR (*"optimi*"* W/2 *"care"*) OR TITLE-ABS-KEY (*"optimi*ation"*) OR (*"organi*ational"* W/1 *"model*"*) OR ((*"policy"* OR *"policies"*) W/2 *"care"*) OR (*"practi*e"* W/1 *"parameter*"*) OR (*"practi*e"* W/1 *"pattern*"*) OR (*"practi*e"* W/2 (*"protocol*"* OR *"policy"* OR *"policies"* OR *"guideline*"*)) OR (*"proactive*"* W/2 *"care"*) OR (*"process*"* W/2 (*"chart*"* OR *"diagram*"* OR *"flowchart*"*)) OR ((*"program"* OR *"programme"*) *"adj project*"*) OR ((*"rule"* OR *"rules"*) W/2 *"care"*) OR (*"standard*"* W/2 *"practi*e*"*) OR TITLE-ABS-KEY (*"surgical pathway*"*) OR (*"treat*"* W/2 *"protocol*"*) OR (*"treatment"* W/2 *"plan*"*) OR (*"treatment*"* W/2 (*"path"* OR *"paths"* OR *"pathway"* OR *"pathways"*)))) AND ((INDEXTERMS (*"Anesthesia AND Analgesia"*) OR INDEXTERMS (*"Anesthesia Recovery Period"*) OR INDEXTERMS (*"Anesthesia"*) OR INDEXTERMS (*"anesthesiologists"*) OR INDEXTERMS (*"Anesthesiology"*) OR INDEXTERMS (*"Anesthetics"*) OR INDEXTERMS (*"Intraoperative care"*) OR INDEXTERMS (*"Intraoperative Period"*) OR INDEXTERMS (*"Perioperative Care"*) OR INDEXTERMS (*"Perioperative Nursing"*) OR INDEXTERMS (*"Perioperative Period"*) OR INDEXTERMS (*"Postoperative care"*) OR INDEXTERMS (*"Postoperative Complications"*) OR INDEXTERMS (*"Preoperative care"*) OR INDEXTERMS (*"Specialties, Surgical"*) OR INDEXTERMS (*"Surgeons"*) OR INDEXTERMS (*"Surgical Procedures, Operative"*) OR INDEXTERMS (*"Operating Rooms"*) OR INDEXTERMS (*"Operative Time"*) OR INDEXTERMS (*"Perioperative Nursing"*) OR INDEXTERMS (*"Preanesthetic Medication"*) OR INDEXTERMS (*"Preoperative Period"*) OR (*"after"* W/6 (*"surgery"* OR *"surgeries"* OR *"surgical*"*)) OR (*"before"* W/2 *"operat*"*) OR (*"before"* W/3 *"procedur*"*) OR (*"before"* W/6 (*"surgery"* OR *"surgeries"* OR *"surgical*"*)) OR (*"during"* W/6 (*"surgery"* OR *"surgeries"* OR *"surgical*"*)) OR (*"follow*"* W/6 (*"surgery"* OR *"surgeries"* OR *"surgical*"*)) OR (*"operating"* W/2 *"room*"*) OR (*"operating"* W/2 *"suite*"*) OR (*"operating"* W/2 *"theater*"*) OR (*"operating"* W/2 *"theatre*"*) OR (*"operating"* W/2 *"unit*"*) OR (*"prior"* W/3 *"operat*"*) OR (*"prior"* W/3 *"surgery"*) OR (*"prior"* W/3 *"procedur*"*) OR (*"undergo*"* W/6 (*"surgery"* OR *"surgeries"* OR *"surgical*"*)) OR TITLE-ABS-KEY (*"an*esth*"*) OR TITLE-ABS-KEY (*"intraoperat*"*) OR TITLE-ABS-KEY (*"intra-operat*"*) OR TITLE-ABS-KEY (*"operation*"*) OR TITLE-ABS-KEY (*"operative*"*) OR TITLE-ABS-KEY (*"peri*procedur*"*) OR TITLE-ABS-KEY (*"perioperat*"*) OR TITLE-ABS-KEY (*"peri-operat*"*) OR TITLE-ABS-KEY (*"periprocedur*"*) OR TITLE-ABS-KEY (*"peri-procedur*"*) OR TITLE-ABS-KEY (*"peroperat*"*) OR TITLE-ABS-KEY (*"pos*ostomy"*) OR TITLE-ABS-KEY (*"pos*otomy"*) OR TITLE-ABS-KEY (*"post*ectomies"*) OR TITLE-ABS-KEY (*"post*ectomy"*) OR TITLE-ABS-KEY (*"post*otomies"*) OR TITLE-ABS-KEY (*"post*otomy"*) OR TITLE-ABS-KEY (*"post*surger*"*) OR TITLE-ABS-KEY (*"post-intervention*"*) OR TITLE-ABS-KEY (*"postoperat*"*) OR TITLE-ABS-KEY (*"post-operat*"*) OR TITLE-ABS-KEY (*"postproced*"*) OR TITLE-ABS-KEY (*"post-proced*"*) OR TITLE-ABS-KEY (*"postsurgical*"*) OR TITLE-ABS-KEY (*"post-surgical*"*) OR TITLE-ABS-KEY (*"preintervention*"*) OR TITLE-ABS-KEY (*"pre-intervention*"*) OR TITLE-ABS-KEY (*"preoperat*"*) OR TITLE-ABS-KEY (*"pre-operat*"*) OR TITLE-ABS-KEY (*"preprocedur*"*) OR TITLE-ABS-KEY (*"pre-procedur*"*) OR TITLE-ABS-KEY (*"reoperat*"*) OR TITLE-ABS-KEY (*"re-operat*"*) OR TITLE-ABS-KEY (*"re-resect*"*) OR TITLE-ABS-KEY (*"resect*"*)))) AND NOT (INDEX (*medline*)) AND (LIMIT-TO (PUBYEAR , *2020*) OR LIMIT-TO (PUBYEAR , *2019*) OR LIMIT-TO (PUBYEAR , *2018*) OR LIMIT-TO (PUBYEAR , *2017*) OR LIMIT-TO (PUBYEAR , *2016*) OR LIMIT-TO (PUBYEAR , *2015*) OR LIMIT-TO (PUBYEAR , *2014*) OR LIMIT-TO (PUBYEAR , *2013*) OR LIMIT-TO (PUBYEAR , *2012*) OR LIMIT-TO (PUBYEAR , *2011*) OR LIMIT-TO (PUBYEAR , *2010*)) AND (LIMIT-TO (LANGUAGE , *"English"*)) AND (LIMIT-TO (SRCTYPE , *"j"*)) AND (LIMIT-TO (SUBJAREA , *"MEDI"*) OR LIMIT-TO (SUBJAREA , *"NEUR"*) OR LIMIT-TO (SUBJAREA , *"MULT"*) OR LIMIT-TO (SUBJAREA , *"PHAR"*) OR LIMIT-TO (SUBJAREA , *"HEAL"*) OR LIMIT-TO (SUBJAREA , *"NURS"*) OR LIMIT-TO (SUBJAREA , *"PSYC"*) OR LIMIT-TO (SUBJAREA , *"DENT"*)) AND (LIMIT-TO (EXACTKEYWORD , *"Aged"*) OR LIMIT-TO (EXACTKEYWORD , *"Middle Aged"*) OR LIMIT-TO (EXACTKEYWORD , *"Very Elderly"*) OR LIMIT-TO (EXACTKEYWORD , *"High Risk Patient"*) OR LIMIT-TO (EXACTKEYWORD , *"Aged, 80 And Over"*) OR LIMIT-TO (EXACTKEYWORD , *"Patient Selection"*))

ClinicalTrials.Gov

79 Studiesfound for: **"perioperative care" | Active, not recruiting, Completed, Suspended, Terminated, Withdrawn, Unknown status Studies | Surgery | Older Adult**
Also searched for **Surgical**. See Search Details

Applied Filters: ☑ **Active not recruiting** ☑ **Completed** ☑ **Suspended** ☑ **Terminated** ☑ **Withdrawn** ☑ **Unknown status** ☑ **Older Adult (65+)**

https://clinicaltrials.gov/ct2/results?cond=Surgery&term=%22perioperative+care%22&cntry=&state=&city=&dist=&recrs=d&recrs=e&recrs=g&recrs=h&recrs=i&recrs=m&age=2

WHO ICTRP

69 records for **66 trials** found for: perioperative care AND surgery
